# Regenerative Therapy of Type 1 Diabetes Mellitus: From Pancreatic Islet Transplantation to Mesenchymal Stem Cells

**DOI:** 10.1155/2016/3764681

**Published:** 2016-03-07

**Authors:** Nadine E. Rekittke, Meidjie Ang, Divya Rawat, Rahul Khatri, Thomas Linn

**Affiliations:** Clinical Research Unit, Zentrum für Innere Medizin, Fachbereich Medizin, Justus Liebig Universität Giessen, 35392 Giessen, Germany

## Abstract

Type 1 diabetes is an autoimmune disease resulting in the permanent destruction of pancreatic islets. Islet transplantation to portal vein provides an approach to compensate for loss of insulin producing cells. Clinical trials demonstrated that even partial islet graft function reduces severe hypoglycemic events in patients. However, therapeutic impact is restrained due to shortage of pancreas organ donors and instant inflammation occurring in the hepatic environment of the graft. We summarize on what is known about regenerative therapy in type 1 diabetes focusing on pancreatic islet transplantation and new avenues of cell substitution. Metabolic pathways and energy production of transplanted cells are required to be balanced and protection from inflammation in their intravascular bed is desired. Mesenchymal stem cells (MSCs) have anti-inflammatory features, and so they are interesting as a therapy for type 1 diabetes. Recently, they were reported to reduce hyperglycemia in diabetic rodents, and they were even discussed as being turned into endodermal or pancreatic progenitor cells. MSCs are recognized to meet the demand of an individual therapy not raising the concerns of embryonic or induced pluripotent stem cells for therapy.

## 1. Clinical Results of Pancreatic Islet Transplantation

Since the introduction of the ground-breaking Edmonton protocol in 1999 [1], pancreatic islet transplantation has become more common treatment for individuals with type 1 diabetes mellitus (T1DM) suffering from recurrent severe hypoglycemia or glycemic lability. Islet transplantation has been associated with limited success during the earlier years, but the clinical results have improved greatly after the Edmonton report [2]. The following section summarizes clinical findings of islet transplantation with focus on metabolic outcomes and diabetic complications in T1DM patients.

### 1.1. Metabolic Outcomes: Glycemic Control and Hypoglycemia

Adult patients included in the islet transplantation process usually have T1DM for more than 5 years, have no preserved endogenous insulin production with negative stimulated C-peptide levels (<0.3 ng/mL), and are prone to severe hypoglycemic episodes or exhibit glycemic instability despite adequate insulin therapy [3]. Hypoglycemia unawareness results often from intensified insulin treatment and is considered the major eligibility criterion for islet transplantation in T1DM patients [4].

In the original Edmonton protocol, seven T1DM patients who received a sufficient islet mass from 2 to 3 donor pancreases became insulin independent with normalized glycosylated hemoglobin (HbA_1c_) levels following a median follow-up of one year. All patients were under corticosteroid-free immunosuppressive regimen consisting of sirolimus, low dose tacrolimus, and daclizumab [1]. After this initial report, follow-up studies in 12 and 17 transplanted patients continued to show positive results including significant decreases in fasting and postprandial glucose levels, normalized HbA_1c_ levels, and improved fasting and postmeal C-peptide secretion as well as increased acute insulin responses to arginine and intravenous glucose tolerance test [5, 6]. A subsequent international trial at nine centers confirmed the reproducibility of the Edmonton results in 21 of 36 patients (58%) who attained posttransplant insulin independence [7]. Other centers that initialized islet transplantation program and adapted the protocol demonstrated comparable outcomes [8, 9]. However, most islet transplant patients returned to insulin injections after a five-year follow-up in Edmonton center. Only ~10% of 65 patients maintained insulin independence, although ~80% remained C-peptide positive. The HbA_1c_ level was nevertheless well controlled in those with partial graft function but increased in those without functioning graft (C-peptide negative). By contrast, hypoglycemic events which were quantified by hypoglycemic scores (HYPO scores) [10] remained significantly improved during the 4-year posttransplant [11], suggesting that even a partial graft function can prevent hypoglycemia and stabilize glycemic control.

Several studies have attempted to refine the Edmonton protocol for achieving and maintaining sustained long-term insulin independence, enhancing islet engraftment, and particularly reducing requirement for multiple islet donors. In 2005, Hering et al. demonstrated restoration of insulin independence following transplantation of islet derived from only a single donor in all eight patients who underwent new immunosuppressive treatment including T-cell depleting antibody (TCDAb) antithymocyte globulin, tumor necrosis factor-alpha inhibitor (TNF-alpha-i) etanercept, and mycophenolate mofetil [12]. A few years later, the same group published a slightly modified protocol using a different maintenance immunosuppression (cyclosporine and everolimus) while retaining the induction therapy (antithymocyte globulin and etanercept) and demonstrated a prolonged insulin independence for a mean of 3.4 years following transplant in four recipients [13]. A more recent study by the same authors reported promising five-year insulin independence rates in patients (50%) receiving induction drugs either with anti-CD3 monoclonal antibody or with the combination of TCDAb and TNF-alpha-i, regardless of maintenance immunosuppression [14]. Similarly, other studies have also applied various immunosuppressive regimens [15–18] and used human islet culture for maximizing islet yield at isolation, ensuring its quality of preparation, and decreasing immunogenicity of allograft tissue [15, 16]. The University of Illinois at Chicago demonstrated recently 60% insulin independence rates in a five-year follow-up trial using immunosuppressive agents etanercept and exenatide without TCDAb. Exenatide, a glucagon-like peptide-1 analog, has the potential to maintain islet survival [18]. Together, all attempts aimed to improve immunologic factors such as alloimmune rejection, autoimmune recurrence, and immunosuppressive drug toxicity as well as nonimmunologic factors including exhaustion of marginal *β*-cell mass [14, 18], all of which have been proposed to cause waning of insulin independence over time. Interestingly, HbA_1c_ was retained within an optimal range (<7.2%) for a 5-year follow-up period in 10 islet recipients irrespective of achieving insulin independence [19].

The most comprehensive results of islet transplantation activity in the last decade were provided by the Collaborative Islet Transplant Registry (CITR). The CITR has collected islet transplant data since 1999 from multiple centers including USA, Canada, Europe, and Australia. At a 3-year interval posttransplant analysis of 677 islet-alone or islet-after-kidney recipients, insulin independence was reported to increase significantly from 27% during the period 1999–2002 to 37% and 44%, respectively, for the years 2003–2006 and 2007–2010. Fasting C-peptide levels decreased less steeply over time, indicating a durability improvement of graft function in the later years. The percentage of patients with HbA_1c_ levels less than 6.5% or a drop by 2% increased accordingly. Additionally, severe hypoglycemic events, defined if patients require outside assistance, were almost abolished (>90% of patients) during the 5-year follow-up of each interval. Overall, metabolic outcomes in patients receiving islet transplantation in 2007–2010 were improved compared with those in 1999–2006 [20]. A number of advancements in transplantation procedure contribute to improved results, including donor selection, islet isolation, islet culture, and peritransplant management, as well as modification in immunosuppression therapy [14, 20, 21].

Intrahepatic islet transplantation has been consistently shown to reduce [11, 16, 22] or even give full protection from hypoglycemia or severe hypoglycemic episodes [1, 5–9, 12–15, 17, 19] and restore hypoglycemia counterregulation in T1DM patients [23–25]. Severe hypoglycemic events can be prevented in islet recipients while C-peptide remained positive but may recur in those with failed grafts [16, 22] albeit less frequent than those prior to transplantation [26]. Hypoglycemia counterregulatory hormones including glucagon, epinephrine, norepinephrine, cortisol, and growth hormone are usually blunted in T1DM patients but were demonstrated to be restored in islet transplanted recipients [23–25]. Normal suppression of endogenous insulin secretion, improved counterregulatory hormone responses [23–25], and increased endogenous glucose production, as well as decreased systemic and muscle glucose uptake [23] all contribute to improving severe hypoglycemia in islet transplanted T1DM patients. Moreover, islet transplantation can ameliorate insulin sensitivity at both liver and peripheral sites as assessed using hyperinsulinemic-euglycemic clamps in 12 T1DM patients [27]. Fear of hypoglycemia, which was quantified using hypoglycemia fear survey, was reduced substantially in T1DM patients following a single islet infusion and was further improved with subsequent infusions [28], confirming the benefit of islet transplantation to abrogate hypoglycemia.

### 1.2. Secondary Diabetic Complications

Long-term near-normalization of blood glucose levels has been reported to significantly delay the progression of microvascular complications including retinopathy, nephropathy, and neuropathy in T1DM [29–31]. Numerous studies have demonstrated stabilized glycemic control following islet transplantation in T1DM patients [1, 5–7, 9, 11–19, 22]. Thus, it appears that islet transplantation may reduce the risk of developing secondary T1DM complications. However, contradictory results exist concerning renal function; some of those studies found elevated creatinine levels, reduced glomerular filtration rate (GFR), and increased albuminuria in islet recipient subjects [5–7, 11, 13, 15–17], while others did not confirm deteriorated kidney function [1, 12, 18]. Moreover, one study demonstrated improvement of kidney graft survival rates, restoration of Na^+^/K^+^-ATPase activity, reduction of natriuresis, and stable urinary albumin excretion in T1DM patients with islet-kidney transplants [32]. Further improvements such as stable creatinine levels and reduced renal resistance index were observed in 24 islet-kidney recipients compared to those with kidney transplant only [33]. By contrast, three other studies showed renal impairment in both islet-kidney and islet-transplant-alone patients, particularly in those with preexisting defect [34–36]. Possible explanations for these observations could be drug-related side effects of sirolimus and tacrolimus [11, 17, 35, 36] or different baseline kidney function prior to islet transplantation [36]. Notably, no evidence of worsening renal function [37, 38] but a slower decline in GFR was found in T1DM patients following islet transplantation compared to control subjects receiving medical treatment [39], indicating less progression of diabetic nephropathy after islet transplantation.

With regard to the effects of islet transplantation on retina function, most studies generally observed a stabilization of retinopathy. In a 2-year follow-up of eight islet recipients, one patient experienced improvement from mild retinopathy to disappearance of the disease at one year and the other seven patients retained pretransplant retinopathy status throughout the follow-up period [40]. In another study, a significant increase of arterial and venous retinal blood flow velocities was found after one year in 10 islet-transplant-alone patients [41]. Further studies consistently demonstrated reduced progression of retinopathy in T1DM patients receiving islet infusion compared to those treated with intensive medical therapy [38, 39, 42].

Islet transplantation may slow the development and progression of diabetic neuropathy. Both sensory and motor nerve conduction velocities (NCV) remained stable in a 2-year posttransplant follow-up of eight patients [40]. Similarly, no significant differences of NCV were observed in islet transplanted subjects compared to control patients receiving medical therapy after a follow-up of one [38] and six years [39], without any changes from baseline in both groups [38, 39]. A significant improvement in NCV was noticed in 18 patients who received islet-after-kidney transplantation for a 4-year period (versus baseline), albeit not different compared to nine control subjects without islet transplantation [43]. Interestingly, a recent study demonstrated an overall amelioration of sensory parameters in 21 T1DM patients over a 5-year posttransplantation period [44], indicating a potential benefit of islet transplantation for the prevention of neuropathy.

Islet transplantation may have positive effects to slow down macrovascular complications. Several studies have demonstrated improvements in vascular function in islet-kidney recipients, including stabilized intima media thickness, decreased signs of endothelial injuring at skin biopsy, increased endothelial-dependent dilation, restored nitric oxide production, and improved atherothrombotic risk factors with elevated levels of natural anticoagulant protein [45, 46]. An overall improvement in cardiovascular parameters was subsequently reported in T1DM patients with end-stage renal disease who received kidney and functioning islet transplants compared to those without islet transplantation or functioning graft [47]. Finally, a 15-month follow-up showed near-normalization of hemostatic and cerebral abnormalities in 12 islet recipient subjects [48].

Thus islet transplantation is principally effective in clinical studies and in different health care systems. However, there are concerns that imply finding alternative technologies as substitute for cadaveric pancreas (Box 1).

## 2. Unsolved Issues of Human Islet Transplantation

### 2.1. Blood Mediated Inflammation at Intravascular Injection

Multiple transplants are required to achieve significant reduction of insulin shots because a thrombotic reaction occurs immediately when isolated islets are exposed to ABO-compatible blood [58]. The reaction is termed Instant Blood Mediated Inflammatory Reaction (IBMIR) and platelets are suggested to take an active part in it. IBMIR further comprises complement getting started and thrombus formed. Blood clots entrapping islets are believed to cause impairment of insulin production of the transplanted islets due to shutting them off from oxygen and to attracting immunocytes [59]. Platelets aggregated in blood clots are recognized to be involved in IBMIR, cellular matrix organization, and cell proliferation and angiogenesis [60].

*In vivo*, platelets get activated if any damage occurs in a blood vessel. They play an essential role in responding to injury that involves the process of haemostasis, thrombus formation, and vascular and connective tissue healing [70]. Platelets come in contact with damaged or disrupted endothelium of the vessel wall and change their shape as they adhere to the damaged site. Collagen is part of the basal membrane of endothelial cells exposed to platelets at sites of vascular injury. Glycoproteins GPVI and GPIIB/IIIa are collagen receptors on platelet surface. Adherence to collagen IV in particular is followed by release of granules containing ADP and TXA which amplify platelet aggregation [71, 72]. Pancreatic islets isolated with collagenase and injected into the portal vein are assumed to include a sufficient quantity of collagen residues to elicit platelet adhesion. GPVI glycoprotein is a strong mediator of platelet-collagen interaction which is associated with FcR gamma chain coreceptor in human and mouse platelets [73, 74]. Furthermore, secretion of inflammatory cytokines IL-1beta, TNF-alpha, IFN-gamma and secondary agonist, and platelet activating factor (PAF) as well as NO production by immune cells and hepatocytes was observed after islet transplantation [75]. These factors reinforce collagen-induced platelet activation and inflammation resulting in positive feedback and causing instability of the graft [76, 77]. It was observed that CD11b and GR1 positive cells were created in the transplantation, promoting inflammatory cytokines production, and cause early graft loss [78].

### 2.2. Organ Quality and Standardized Mass Isolation of Islets

**Figure 1:**
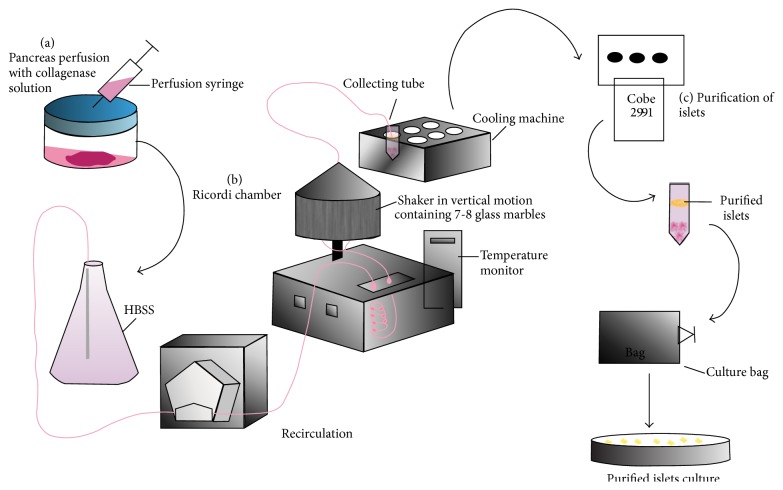
Schematic representation on mass islet isolation from human or pig. (a) Pancreas perfusion with collagenase NB 8. (b) Pancreatic digestion. (c) Purification of islets.

The primary object of isolating human pancreatic islets is for treatment of type 1 diabetic patients by intraportal injection to restore beta-cells functions. Our group has isolated mass numbers of human and pig islets for research purposes (Figure 1). Attention to key factors like enzymatic digestion of pancreatic tissue containing islets, pH of solutions used in isolation, purification of islets, and temperature during isolation is essential for successful islet isolation and culture to maintain viability for mass production [79, 80]. In principle, the resected pancreas is perfused with collagenase solution consisting of collagenase NB 8 (SERVA Electrophoresis, cat. number 17456) and neutral protease via the pancreatic ducts. After filling with collagenase solution pancreas is transferred to Ricordi Chamber (digestion chamber) [79, 81, 82] on top of the 7 to 8 glass marbles under continuous perfusion with HBSS (Biochrom). The aim is to dissolve connective tissue at room temperature and thus to release the islets from exocrine. The chamber is connected with a tubing system to recirculate fresh solution and maintain dissection temperature between 32 and 37°C. Ricordi Chamber is set in vertical motion; shaking is initiated automatically and manually followed by two centrifugation steps at 80 ×g. Digestion progress is monitored by taking samples repeatedly to identify number and size of isolated islets. Furthermore, islets are purified by centrifugation with a Cobe 2991 using a continuous HBSS-Ficoll gradient. Purified islets fractions are finally cultured either in CMRL 1066 or in RPMI 1640, with supplement including antibiotic and serum at 37°C [83, 84]. Prolonged cultivation will enhance survival and functional quality of transplanted islets by reducing IBMIR [85, 86].

In the long run, sources of pancreatic beta-cells should be investigated which are more flexible and circumvent all of the disadvantages delineated in Box 1. Recently, pluripotent embryonic stem cells have been extensively studied, but there are major ethical issues. Moreover, teratoma formation prevented clinical studies up to now. One of the more promising options is the use of multipotent adult stem cells because they can be retrieved from the same individual, they are less prone to malignant transformation compared to embryonic stem cells (ESC), and clinical trials in diabetes treatment have successfully been initiated [87, 88].

## 3. Mesenchymal Stem Cells

Mesenchymal stem cells (MSCs) are nonhematopoietic, multipotent, self-renewing cells. First they were isolated and described from bone marrow in 1968 and described as adherent, spindle-shaped, and with the ability to differentiate into bone and cartilage [89]. They were described as stromal cells [90, 91] and later also as mesenchymal stem cells [92]. Since then it was discovered that mesenchymal stem cells can originate from a wide range of different tissues such as skeletal muscle [93], skin and foreskin [94, 95], adipose tissue (AD-MSC or AT-MSC or ASCs) [96], pancreas (P-MSCs) [97], dental pulp (DPSCs) [98], salivary gland [99], endometrium [100], placenta (PL-MSCs) [101], amniotic membrane and fluid (AM-MSCs) [102–105], umbilical cord matrix/umbilical cord blood (UC-MSCs/UCB-MSCs) [106, 107], and Wharton's jelly (WT-MSCs) [108].

First it was thought that MSCs could only differentiate into somatic cells of the same germ layer, but they have shown a much higher level of plasticity and are able to differentiate* in vitro* across germinal boundaries. To date, MSCs have been shown to differentiate* in vitro* into adipocytes, chondrocytes, osteoblasts, myoblasts, tenocytes, cardiomyocytes, marrow stromal cells, hepatocytes, endothelial cells, hematopoietic cells, neuronal cells, renal cells, and pancreatic cells [49, 96, 109–120].

According to the International Society for Cellular Therapy (ISCT), the standard criteria for all human MSC are threefold: plastic adherence under standard culture conditions; a positive phenotype (≥95%) for CD105, CD73, and CD90; and a negative phenotype (≤2%) for CD45, CD34, CD14, or CD11b; CD79alpha or CD19; HLA-DR; they must be able to differentiate to adipocytes, chondroblasts, and osteoblasts under standard* in vitro* culture conditions, demonstrated by* in vitro* staining. As MSCs have to be isolated from surrounding tissue and infiltrating cell types, like immune cells, blood cells, and endothelial cells, negative phenotype antigens were selected to exclude these other cell types. To determine the state of stimulation of MSCs, the surface marker HLA-DR is chosen. Unstimulated, HLA-DR is not expressed but, after stimulation, for example, with IFN-gamma, HLA-DR is expressed on the cell surface [121, 122].

## 4. Bone Marrow Mesenchymal Stem Cells (BM-MSCs) and Adipose Tissue Derived Mesenchymal Stem Cells (AT-MSCs, ASCs, and AD-MSCs)

BM-MSCs and AD-MSCs are very similar in their expression pattern of surface markers; both express the marker which identifies them as MSC according to the ISCT guidelines as described before. Accordingly, both cell types are positive for CD105, CD73, and CD90 [121]. Several research groups published new markers for MSC and propose that these should be added to the list of suitable markers to describe them, like STRO-1 [123, 124], STRO-3 [125], CD56, CD271, mesenchymal stem cell antigen-1 (MSCA-1) [126, 127], and CD166 (ALCAM) [109]. Additionally, BM-MSCs and AD-MSCs both show expression of CD177 (stem cell factor receptor) [128], CD29 (beta-1 integrin), CD44 (hyaluronate receptor), CD49e (alpha-5 integrin, important for cell adhesion to fibronectin), CD146 [109], CD9, CD10, CD13, and CD59 [129].

Although the patterns of surface markers expressed from AD-MSCs and BM-MSCs are very similar, there are some differences: BM-MSCs are positive for CD106 (VCAM-1), which lacks in AD-MSCs, and they are negative for CD49d (VLA-4), which is strongly expressed in AD-MSCs [130, 131]. Interestingly, CD106 is the receptor for the related agonist CD49d and these molecules are involved in hematopoietic stem and progenitor cell homing to and mobilization from the bone marrow [132, 133]. Both express CD54 (ICAM-1), but AD-MSCs are in higher levels compared to BM-MSCs [130]. AD-MSCs can be retrieved in a much higher number and under chirurgical easier conditions with lesser pain for the patient or donor than BM-MSCs [134] and they tend to be genetically more stable in long-term culture [135, 136]. AD-MSCs and BM-MSCs express Nestin and the pancreatic transcription factors ISL-1 and Pax6, which indicates that the use of these for endodermal differentiation may be advantageous, compared to other MSCs [49–51, 69].

## 5. Immunomodulatory/Immunosuppressive Effects of MSCs

### 5.1. MSCs as “Helper” Cells in Transplantation

Transfusion of MSCs in streptozotocin-induced diabetes in mice reduces hyperglycemia and enhances beta-cell function and survival. However, it is not clear whether this is caused by the MSCs themselves or through the release of trophic factors [137, 138].

Cotransplantation of MSCs with pancreatic islets in mice leads to improved islet function and survival. Borg et al. observed improved glucose homeostasis and reduced islet apoptosis after cotransplantation of islets with MSC at three different locations: kidney capsule, liver, and eye. According to the authors, MSCs did not increase beta-cell proliferation and MSC differentiation into pancreatic beta-cells could not be detected [139].

MSCs from various tissues show immunomodulatory and/or immunosuppressive properties. Transplanted or cotransplanted MSCs decrease proliferation and activation of T-cells, dendritic cells, and NK cells in the recipient. They decrease the secretion of inflammatory cytokines, like IFN-gamma, TNF-alpha, GM-CSF, and MCP-1. Additionally, they act as immunomodulator and help in the establishment of a graft vascular network by secreting angiogenic paracrine factors as VEGF, IL-6, IL-8, HGF, PDGF, and TGF beta and the secretion of matrix metalloproteinases. Furthermore, TGF beta, HGF, and IL-6 have antiapoptotic effects and increase the expression of protective genes against hypoxia [140–143].

## 6. Endocrine Pancreas Cell Lineage

**Figure 2:**
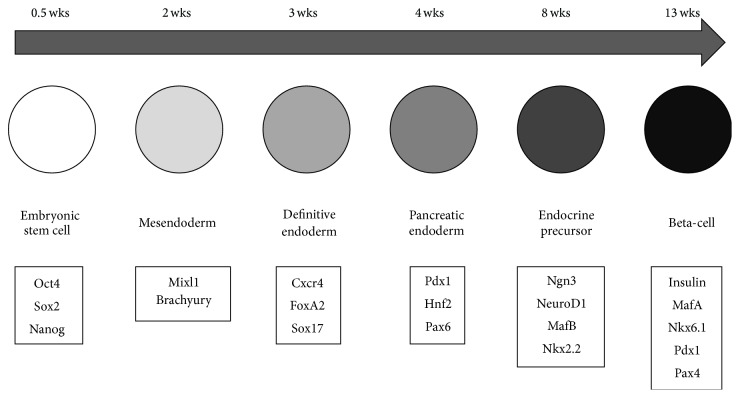
Beta-cell development-scheme in human embryo.

Pancreatic development is a highly regulated and complex process, which is guided by multiple signaling pathways and transcription factor cascades (Figure 2). First epiblast cells ingress in the primitive streak to form the mesendoderm, characterized by the transcription factors* Mixl1* and* Brachyury*, and then the definitive endoderm [144, 145]. Definitive endoderm cells expressing transcription factors such as Cxcr4, FoxA2, and Sox17 form the gastrointestinal organs, such as liver, lungs, thymus, respiratory and digestive tract, and pancreas [146–148]. They are, however, not committed towards a specific cell or tissue lineage in the initial differentiation. Thus, an important specification step occurs when the definitive endoderm cells form the posterior gut endoderm, which develops subsequently into the intestines [147, 149]. At the foregut-midgut junction, the expression factor Pdx1 is expressed. Pdx1-positive cells were shown to contribute to the formation of exocrine and endocrine compartments of the pancreas [150, 151]. Posterior and anterior foregut endoderm develop into the ventral and dorsal pancreatic buds. In this phase, the interaction with the mesoderm-driven neighboring tissues, cardiac mesenchyme at the ventral bud and notochord at the dorsal bud, regulates pancreas organogenesis and the subsequent specification steps [152]. Involved morphogens are activin A and fibroblast growth factor (FGF) from the notochord and fibroblast growth factor and bone morphogenic proteins from the cardiac mesoderm [148, 151, 153, 154].

After this, the pancreatic buds are formed from multipotent progenitors that contribute to all pancreatic cell types. These epithelial buds invade the surrounding mesenchyme by successive waves of branching morphogenesis called the primary and secondary transition. The period of active pancreatic progenitor proliferation, followed by expansion of the epithelial network, is identified as primary transition. The first endocrine cells are detected at this stage [155, 156]. In the secondary transition, the morphogenetic transformation of pancreatic epithelium occurs. The specification of the multipotent progenitors towards the differentiated lineages occurs in this period. Particularly, the endocrine cell specification and differentiation occur via the inhibition of Notch signaling, which leads to the expression of Ngn3 [157, 158]. This triggers the expression of a multitude of expression factors, including Nkx2.2, NeuroD, Nkx6.1, Pax4, Pax6, and Isl1, controlling endocrine cell differentiation [151, 159]. Afterwards those endocrine cells begin pancreatic islet morphogenesis through aggregation into small cell clusters [160]. The final maturation of the Langerhans islets takes place after birth [161].

Two crucial postnatal maturation events need to occur for fully functional beta-cells: (1) glucose sensing is enhanced and the amount of insulin-containing dense core secretory granules increases. (2) Beta-cell mass appropriate to the individual body weight is established. Several genes encoding important factors are involved in this process of postnatal beta-cell maturation: insulin and preproinsulin; glucose transporter (Glut2) and glucokinase (GK); Pdx1, MafA, and NeuroD (important transcription factors for development and function of mature beta-cells) [151, 162–164]. How the postnatal maturation occurs in detail is still largely unknown [165].

## 7. Differentiation of MSCs via Chemical Compounds

Most chemical differentiation protocols are based, with slight modifications, on the original protocol from Timper et al., 2006. The general approach of the Timper et al. strategy is a protocol consisting of one or two steps aiming to directly differentiate MSCs to insulin-producing cells (IPCs) without following the developmental steps known from embryonic pancreas development. Culture medium contained high glucose concentrations, activin A, nicotinamide, Exendin-4, HGF, and pentagastrin among other supplements (Table 1). Following the procedure, MSCs expressed nestin, SCF, Thy1 (CD90), Pax6, and Isl1. While Isl1, Pdx1 (=Ipf1), and Ngn3 were upregulated, the expression level of Pax6 remained unchanged. Besides insulin, specific for beta-cells, expression of glucagon (alpha-cell) and somatostatin (delta-cell) was reported [49].

**Figure 3:**
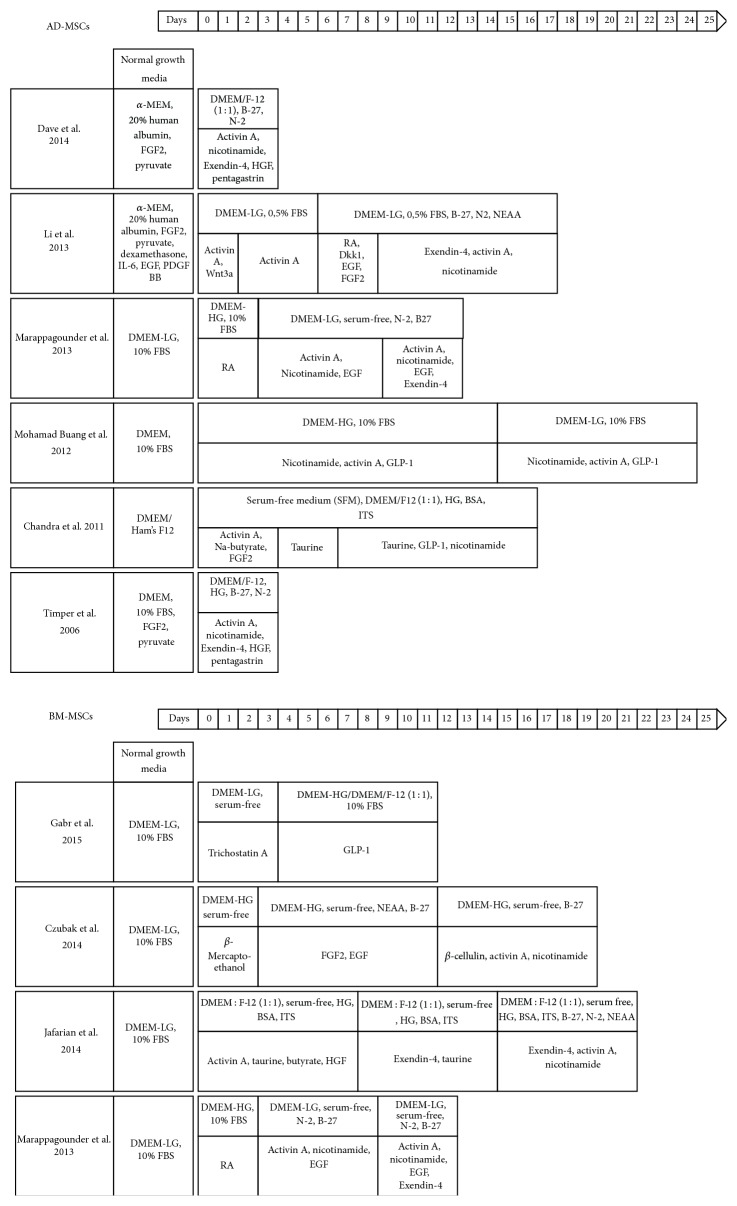
Differentiation of MSCs via chemical compounds. AD-MSCs [49–54] and BM-MSCs [51, 55–57].

Dave et al. adjusted the differentiation process developed by Timper et al. onto a serum-free condition using 20% human albumin instead (Figure 3). The cultured cells were described as positive for Pax6, Isl1, and Pdx1 and they were insulin and C-peptide positive upon glucose stimulation (Table 2). They also proposed that stepwise and long-term culture conditions over several weeks would not be necessary [52]. This would be contradictory to the findings of other groups, as described below.

The second major approach is adapted from protocols for beta-cell differentiation from embryonic stem cells (ESCs), trying to mimic the steps in human pancreas development via a stepwise differentiation. Therefore, the added chemicals are changed every two to three days, accompanied by measuring the expression of typical markers of the expected stage, like definitive endoderm or pancreatic progenitor cells (Figure 3).

Li et al. found an upregulation of Brachyury, Mixl1 (mesendoderm-related genes), FoxA2, Sox17 (DE-related genes), Sox1, and Pax6 (ectoderm-related genes) depending on activin exposure in a dosage dependent manner. They did not find upregulation of mesoderm related genes (Figure 3). Activation of Wnt-signaling in step 1 reduced the expression of ectodermal and favored endodermal genes. To drive DE cells towards pancreatic progenitor (PP) cells the cells were cultivated with a Wnt-signaling inhibitor, retinoic acid (RA), FGF2, and other cytokines. After 6 days, pancreatic endoderm and progenitor cell markers, like Pdx1, Sox9, Hnf6, Nkx2.2, and Nkx6.1, were upregulated and the expression of FoxA2, Sox17 (DE-related genes), and Mixl1 (mesendoderm-related gene) was downregulated. The combined approach of activating RA/FGF signals and inhibiting Wnt signals was reported to provide similar results to differentiation protocols of ESCs and iPS-cells. Is this more evidence that MSCs are a suitable substitute for ESCs? Li et al. studied the differentiation potential of AD-MSC-derived PP cells to further differentiate into downstream endocrine and exocrine pancreatic lineages. They found cooverexpression of insulin (beta-cell-specific), glucagon (alpha-cell-specific), somatostatin (delta-cell-specific), pancreatic polypeptide (PP) (PP-cell-specific), ghrelin (epsilon-cell-specific), MafA, and Glut2. Not all expressed proteins were specific for beta-cells. Rather a potpourri of hormones expressed from immature endocrine islet cells was detected. Still, those immature cells responded upon glucose stimulation with insulin/C-peptide. Moreover, PP cells were shown to be driven towards an exocrine expression pattern [53].

When BM-MSCs were cultured for several weeks up to months in the presence of high glucose concentrations, a differentiation process was promoted initially, but at a later stage low glucose and low serum conditions were beneficial to raise glucose-sensitive IPCs, characterized by Pdx1, Ngn3, Isl1, NeuroD, Pax4, and insulin. Interestingly, IPCs did not overlap with other endocrine cell types. Reduction of hyperglycemia, but unfortunately also tumor formation, was reported [166]. Mohamad Buang et al. [54] chose a two-step protocol over 21 days for AD-MSCs. They found that round cells after two weeks which started to form clusters could be positively stained with dithizone (DTZ) and contained small secretory granules, all characteristics for beta-cells. They released insulin in a glucose responsive manner. Similar to Tang et al. [166], they utilized high glucose followed by low glucose concentrations in the culture media.

One group examined human BM-MSCs and human AD-MSCs in a three-step protocol in parallel. Both cell types could differentiate into IPCs with BM-MSC-derived IPCs appearing to generate more islet-like clusters than those derived from AD-MSCs. The gene expression profile included nestin, Pdx1, Isl-1, Pax4, and Ngn3 and was similar in both cell types, also showing glucose dependent insulin release (Table 2). MSCs from both sources seem to be useful for beta-cell replacement strategies [51]. The authors concluded that BM-MSCs are more feasible for MSC differentiation towards IPCs than AD-MSCs. However, due to the expression profiles of both experiments being nearly identical to each other, this conclusion seems rather premature and optimistic and is in need of conformation.

The advantage of a stepwise procedure is the possibility of obtaining progenitor cells first, which can be directed to pancreatic cell types in the last step. For example, recent studies postulate that Wnt- and BMP-signaling are relevant for differentiating MSCs indicating that MSCs are directed towards beta-cell differentiation like iPS cells. Therefore, in early differentiation steps, Wnt should be activated but in later stages inhibited. In stepwise differentiation protocols, the signaling pathways can be better controlled, postulating an advantage of stepwise differentiation protocols over one-step protocols [53].

How to reproduce competent pancreatic progenitors is actually under debate. There are no consensus reagents or standard protocols available to characterize progenitor cell lines. Moreover, it is not clear if differentiation of MSCs should undergo the same stages as embryonic cells/embryonic stem cells. Whether MSCs can fully transdifferentiate into cells of other germ layers is under discussion, because developmental biologists have restricted the definition of transdifferentiation to an irreversible switch of one cell type to another [167]. In several studies, MSCs showed a new expression pattern of formerly not expressed genes specific to the new somatic cell type; on the other hand they still express MSC specific markers, like CD90 and CD73. Given these results, transdifferentiation of MSC may need to be reevaluated.

## 8. Differentiation of MSCs via Genetic Manipulation

Experiments with transgenic mice have identified essential transcription factors that control the formation of islets during embryonic development; among these are FoxA2 (formerly named HNF3beta), PDX1 (IPF1), NGN3 (NeuroG3), NeuroD1, Nkx2.2, Pax4, Nkx6.1, and Pax6. Among these factors, FoxA2 and PDX1 play a central role in initiating the differentiation of the beta-cells.

Several research groups induced differentiation in MSCs through overexpression of genes involved in the beta-cell development and beta-cell function, either by transduction with viral vectors, like lentivirus or adenovirus, or by transfection with plasmids. Some tried PDX1 gene alone and others in combination with NGN3 or NGN3 and MafA. Only few groups tried other genes like FoxA2 or HLXB9 (Table 3).

In the studies, which induced differentiation through the selective overexpression of PDX1, several key transcription factors are increased by exogenous PDX1, like FoxA2, Nkx2.2, and NeuroD (Table 3). However, FoxA2 is not specific for pancreas development; it also plays a role in the hepatic lineage. Lee et al. changed the medium to a high glucose containing one for up to 4 weeks, when over 60% of the cells were PDX1-positive following infection. During this time period, PDX1-induced cells began to form clusters [63]. Allahverdi et al. also transduced Pdx1 but did not change the medium to high glucose [67]. All groups reported morphological alterations after 3 to 5 days and cluster formation after 7 or 14 days [63, 65, 67]. At the same time, cell proliferation slowed down. Lee at al. and Lin et al. found FoxA2, Nkx2.2, and NeuroD at low basal expression levels in nontransduced control cells and upregulated in PDX1-overexpressing cells under high glucose conditions. Additionally, GLP-1, glucokinase (GK), and GLUT2 were expressed at low level with PDX1 induction. Mouse PDX1 overexpression induces the expression of the human homolog PDX1 (=IPF1). It was concluded that PDX1 was able to upregulate its own expression [63]. In contrast, Karnieli et al. did not find activation of the human Pdx1 gene through rat Pdx1 overexpression [69]. Transplantation of PDX1-overexpressing hAD-MSCs into diabetic rodents reduced blood glucose levels and prevented severe hyperglycemic states but did not achieve euglycemia. The transplanted cells stained positive for insulin [63, 65]. Karnieli et al. did not find Ngn3 expression and only found NeuroD1 expression after transplantation of Pdx1^+^ MSCs in STZ-induced diabetic mice. They postulated a maturation process* in vivo*. Allahverdi et al. measured Pdx1 and insulin and detected a higher expression of Pdx1 not earlier than day 7 and insulin not before day 14 with maximum expression of both at day 21 after transplantation. Insulin and C-peptide levels stimulated by glucose were increased in recipients of transplants [67]. As they did not compare insulin and C-peptide secretion to nondiabetic individuals, there is no conclusion whether the quantity of released insulin was biologically significant. Karnieli et al. reported that insulin release after glucose challenge was clearly subnormal [69].

Two studies directly compared insulin release of manipulated MSCs through genetic manipulation, chemical compounds, and conditioned medium with human islets and/or cocultured human islets [68, 168]. Moriscot et al. compared different infection rates of the adenovirus, different transcription genes PDX1, FoxA2, and/or HLXB9. Transduction was combined with conditioned medium from pancreatic islets or chemical compounds. They observed different transcription factor patterns, depending on the virus titer. If the multiplicity of infection (MOI) ratio was high, insulin would be released from the cells. On the other hand, coculture or conditioned medium from pancreatic islets was additionally needed with low MOIs. Some of their protocols resulted in Pax4, Isl1, NGN3, and insulin expression, while in others only Pax4 or Pax4 and low insulin were expressed [168].

Limbert et al. compared chemical differentiation with genetic manipulated differentiation. One very important finding was that chemical differentiation leads to clustered cells with decreasing proliferation till the cells died after two weeks in culture. Cells induced by chemicals expressed Pax4, Isl1, Pdx1, NeuroD, somatostatin, pancreatic polypeptide (PP), insulin, and Glut2 but neither Ngn3 nor glucagon. Pax6 was detected in untreated and treated MSCs as well. In contrast to chemical induction, Pdx1, Ngn3, and Pdx1-Ngn3-cooverexpression did not result in distinct islet-like clusters in the genetically treated cells. Combined overexpression showed induction of Pax4, Isl1, GLU2, insulin, glucagon, PP, and somatostatin. Pax6 expression was also upregulated. C-peptide and insulin expression remained below biologically significant levels and as the cells also expressed non-beta-cell hormones, a further maturation of these cells was desired [68].

Boroujeni and Aleyasin combined exogenous Pdx1 lentiviral expression with high glucose medium containing additional compounds, nicotinamide, and bFGF for 21 days. After 7–10 days of culture, Pdx1^+^ MSCs changed their morphology and formed clusters. Higher expression of Pdx1, Ngn3, Glut2, and somatostatin was detected. After transplantation of these cells in diabetic mice, hyperglycemia was reverted [64].

Recently Bahrebar et al. combined Pdx1 transfection with cultivation under high glucose condition till islet-like clusters were formed after 10 days of cultivation. Pdx1, Ngn3, Nkx2.2, and insulin gene expressions were measured in the group transduced with Pdx1 [62]. Insulin gene activation was glucose responsive and it was concluded that cultivation of Pdx1^+^ MSCs in high glucose was beneficial to the maturation of these cells towards a beta-cell phenotype [54]. Posttransplant maturation of* in vitro* differentiated MSCs was claimed [56, 65, 69]. By contrast, spontaneous* in vivo* maturation without prior specific cultivation protocol was denied [169].

Jafarian et al. utilized microRNAs (miRNAs) to differentiate MSCs towards insulin-producing cells. Several miRNAs have potential roles in pancreas development, islet function, and insulin secretion. miR-375 was involved in pancreas development and control of insulin gene expression. miR-9 downregulated insulin expression and was described as a negative regulator for insulin-producing cells [170–173]. MSCs were transduced with miR-375 and anti-miR-9. The morphology of the MSCs changed within 3 days from spindle-like to round cells and in the next 14 days cell clusters formed. The clusters showed gene upregulation of Sox17, FoxA2, Nkx2.2, Glut2, Pdx1, Ngn3, insulin, PP, and somatostatin (Table 3). Cells cotransduced with miR-375 and anti-miR-9 proved to be insulin glucose dependent and therefore they showed synergistic effects in MSC differentiation to insulin-producing cells [66].

Most groups agree that reduction of hyperglycemia via transplantation of differentiated MSCs should be further investigated to elucidate the underlying mechanisms. MSCs do not strictly possess beta-cell characteristics prior to transplantation as only part of the genes important for beta-cell function are detectable. Other issues are first the number of cells that are required to significantly reduce blood glucose and second how to improve the condition of the cells at the time of transplantation to increase their* in vivo* viability [65].

## 9. Prerequisites for Clinical Use of Insulin-Producing Cells Derived from Differentiated MSCs

In spite of a lot of different approaches to differentiate MSCs towards insulin-producing cells and trials toward the curing potential of IPCs in animal models, there are still some issues which are not yet addressed but are crucial for clinical application. The outstanding concerns are, for example, as follows: “Are obtained IPCs stable in* in vivo* conditions or is dedifferentiation an issue?” “Which of the protocols are adaptable to xeno-free, GMP-complaint standard procedures?” “Is it enough to transplant insulin-producing cells, which are only part of functional islets?” What can we learn from islet transplantation and what can be adapted from those approaches to MSCs-derived IPCs? Are the IPCs on the long term stable? What about cell viability subsequent to transplantation?

The following list delineates major prerequisites for clinical use of IPCs derived from MSCs:

1. High purity and quality of cells in terms of bacterial, viral, and mycoplasma contamination; no animal-derived substances including safety tested compounds of animal origin.
2. Culturing, expanding, and biobanking of MSCs under GMP conditions.
3. Quality of* in vitro* generated cells: pancreatic islets as control cells, glucose responsiveness of C-peptide, and/or insulin expression close to physiological conditions.
4. Long-term* in vitro* and* in vivo* stability of  IPCs derived from MSCs.
5. Exact dosage of MSC-derived IPCs to reverse diabetic condition and feasibility of producing such dosage* in vitro*.
6. Improvement of cell survival after transplantation, engraftment, and homing of IPCs and MSCs.
7. Exclusion of tumor risks.

It is of importance to bring the glucose responsiveness and the maturation of the IPCs to a similar level of original beta-cells. At present, glucose dependent insulin release is in most reported studies far below physiological level of beta-cells [68, 69, 168, 174, 175]. If insulin expression and release cannot be significantly raised, transplanted patients will be still dependent on insulin shots. Given transplantation of allogenic, gene-manipulated tissue as such an invasive procedure, the expected outcome does not justify the potential risks. The goal should not only be to ameliorate blood glucose levels and to prevent acute events of diabetic ketoacidosis but to give diabetes patients the chance of normal life [175, 176].

A very important question for the clinical use of MSCs is the risk of tumor formation. There are contradictory reports, some excluding tumors, but others measured changes in karyotype and telomere length, describing the presence of tumor markers, and some groups even reported* in vivo* tumor formation in mice. However, MSC culture conditions potentially leading to tumor formation were not investigated. Unfortunately, in publications observing tumor growth, GMP standards were not reported. Additionally, whether immunosuppressive properties of MSCs cause adverse effects is discussed, such as infections or graft versus host disease. A series of clinical trials using MSCs for treatment purpose is already going on [64, 166, 177–180]. It seems that if MSCs pose a risk of tumor formation, it can be hypothesized to be a lot weaker and perhaps better controllable as in the case of ESCs and iPS cells.

In spite of immunosuppressive properties of MSCs, there is still evidence of graft rejection and impaired cell survival after transplantation which calls for further investigation and leads to the prerequisite that pretransplantation conditions have to be optimized. There is only little data available in this direction of preconditioning the MSCs before transplantation to increase* in vivo* viability, but further research is certainly required.

## 10. *Increase of Survival of MSC* for Transplantation and Clinical Application

After transplantation of MSCs, most of them diminish due to apoptosis [181]. To overcome the current limitation and improve transplantation therapy, pretreatment of MSCs with different modulatory factors is an approach to boost their predefined potential for type 1 diabetes and other disorders.

When MSCs were cultivated at hypoxia or when transplanted* in vivo*, MSCs likewise suffered from low oxygen concentration (0.5% to 2.3%) [182–184]. This change in O_2_ concentration of MSCs may contribute to DNA damage and early senescence [185–187]. However, hypoxia-inducible factor 1 (HIF-1) plays vital role in regulating different gene expression of stem cells in cellular response to hypoxia and pretreatment of MSCs with hypoxia condition can improve engraftment potential [187]. Liu et al. demonstrated increase in angiogenic factor (HIF-1, ANG, VEGF, and MMP-9) and Bcl-2 expression with hypoxic preconditioned MSC (5% O_2_) [188]. Therefore, hypoxia can provide a protective shelter to transplanted MSCs to some extent. Apelin 13 is an endogenous ligand for G protein coupled APJ receptor [189]. Apelin has been involved in maintaining cardiovascular functions and other biological activities [190]. HIF-1 pathway provides protective effect to hypoxic treated MSCs as discussed above. However, hyperglycemia along with hypoxia preconditioned MSCs could produce reactive oxygen species and affect cell integrity [191, 192]. Apelin 13 provides the protective effect from apoptosis via MAPK/ERK1/2 and PI3K/AKT signaling pathways in bone marrow derived mesenchymal stem cells [193]. Mottaghi et al. hypothesized that pretreatment of hypoxic preconditioned mesenchymal stem cells with apelin 13 could provide protective effect against apoptosis in stem cell transplantation therapy by reducing ROS level via MAPK/ERK1/2 and PI3K/AKT signaling pathways [192]. Therefore, hypoxia-MSCs-apelin 13 combination can be considered as one strategy to rescue transplanted cells in diabetic condition.

Pretreatment with modulatory factors can ameliorate the surviving capacity and engraftment properties and have potential to resolve current limitation of MSCs in transplantation therapy. However future strategies and better understanding of unfavorable microenvironment of posttransplanted MSCs could provide a better approach.

## 11. More Efficient Insulin Release and Immunosuppression

In a healthy person, the pancreas consists of 10^9^ cells and a normal insulin release of 1–15 mg per day [81, 194]. As MSC-derived IPCs are far away from clinically relevant insulin concentrations, either the insulin release capacity of each cell has to rise or more IPCs have to be transplanted. Some groups estimate an amount of 10^9^ IPCs to be transplanted per patient, which amounts to the total cell mass of the whole pancreas in IPCs alone [175]. However, most studies report that MSCs stop or slow proliferation in the differentiation process. Therefore it is important to work on the yield of IPCs. At present, all studies are conducted in small-scale flask cultures. An upscale towards a bioreactor-volume needs to be established, but only few groups are working on this end.

## 12. GMP

Progressing cell and tissue manipulation techniques from pure research towards clinical application, that is, cellular or gene therapies, demands an additional framework laid down by regulatory bodies like the FDA or EU called good manufacturing practice (GMP). GMP requires that a therapeutic product is of the highest possible quality and poses no risk for the recipient [195]. Working in compliance to GMP encompasses not only the process for production of the therapeutic product but the whole laboratory as well.

Since stem cell therapies are comparatively new, the corresponding regulatory requirements are still developing. Currently cell therapy requirements are classified according to the degree of manipulation involved and the expected process-related risks. While minimal manipulations, such as cryopreservation of tissues or cells, are regulated under good tissue practices (GTPs), which is already present in most clinical labs, more extensive manipulations, for example, transduction,* ex vivo* expansion, activation, and use for other than the tissues function, fall under the more strict GMP. MSCs are classified as advanced therapy medicinal products per European Regulation No. 1394/2007 and are further considered as somatic therapy or tissue-engineered products, depending on source, manufacturing, and indication. Therefore, production and delivery of MSCs must be carried out in accordance with European regulations [196–201].

The major disparity of GMP cell engineering and the established GMP in the biopharmaceutical industry is that most cell therapies are custom-made to the individual patient whereas biopharmaceutical GMP aims towards bulk production. In an ongoing process adjustment of the regulations is required. However, continuous renewal makes the classification and GMP requirements of cell therapeutic products subject to change. For example, first GMP complaint procedures for cell expansions are already established [202, 203]; in contrast GMP procedures for differentiation are still not defined.

Translating research-based protocols into GMP complaint procedures for the production of clinical-grade MSCs requires an in-depth assessment of all critical aspects and involved risks. All phases of production must be subjected to quality control. This includes, but is not limited to, process controls, which qualify cell production technique, including functional tests, and production controls, for example, bacteriological tests, phenotypic controls, and a visual follow-up. Critical is the analysis that the culture protocol does not lead to cell transformation (karyotype, FISH, quantitative expression of telomerase, c-myc, etc.). Tests of viability and phenotype must be performed at the final stage but must additionally be compatible with a rapid release of the finished product [177].

One of the most crucial aspects for the maintenance of phenotype and genotype of cultured MSCs during multiple passages is the cell culture media. Agreement on the optimal media for MSC cultivation is not reached in present. Most commonly DMEM, MEM, EMEM, *α*-MEM, and RPMI with supplementation of FBS, human serum or plasma, and growth factors are used  [49, 50, 52, 68, 202]. This leaves much room for improvement; apart from the lack of standardized protocols, the use of FBS is undesirable by GMP standards, which favor a xeno-free approach to eliminate the risk of cross-species disease transmission. Furthermore, utilizing FBS in GMP production requires additional certification of the used FBS, significantly raising the costs. First steps towards wholly synthetic culture media are already undertaken, with the aim of eliminating these concerns. Salzig et al. tested a chemical defined culture medium for expansion of MSC and established this for flask cultures up to bioreactor scale. They specify “chemical defined” as being xeno-free as well as free from components derived from or consisting of lysates, hormones, transferrin, or similar compounds, arguing that their composition or activity is susceptible to deviation. This approach is almost unique for MSCs, efforts by other groups research limiting themselves to xeno-free-only culture media until now, which is not chemical defined in the strictest definition [204].

## 13. Concluding Remarks

As diabetes is a disease which strikes millions of people worldwide, which goes along with lifetime necessity of insulin shots and high risk of side effects because the best glucose control is still not comparable with physiological glucose regulation, a beta-cell replacement treatment is highly desirable. The option of transplanting pancreas or isolated islets is limited because of a lack of suitable organs relative to the large amount of potential recipients, combined with severe side effects caused by lifelong immunosuppression, which has to be weighed against the necessity of insulin shots and is therefore only recommendable for a subgroup of patients with severe medical history. The option of xenotransplantation, which would resolve the lack of donors, for example, with pig islets, poses even bigger risks of other adverse effects. Despite these concerns, important knowledge comes from clinical and experimental islet transplantation and it is still one of several treatment options that are worthwhile to follow in the future.

As there are a multitude of approaches in the field of regenerative medicine, ranging from novel ways to induce beta-cell proliferation, reprogramming of other pancreatic cells or nonpancreatic cells like liver cells, the differentiation research on iPSC, fetal stem cells, or adult stem cells (among them, MSCs), it is not possible yet to anticipate which technique(s) will come out on top. Therefore, it is very important that each path is worked on among researchers in the diabetic field. In this review, MSCs are highlighted, because they have great potential and do not come with ethical issues as opposed to ESCs due to the fact that for ESCs embryos have to be destroyed. Additionally, MSCs can possibly act as supporting cells along with classical islet transplantation or ameliorate diabetes by using the MSCs in an undifferentiated status. Some clinical studies are already going on, using MSCs, because of their anti-inflammatory potential.

Preliminary results seem to show that the tumor risk is low to absent in MSCs compared to iPS cells and ESCs. In spite of the fact that research is underway for the use of MSCs in diabetes treatment, clinical application is still a long way to go as there is still a lack of standardized protocols to produce and expand MSCs, to better control the risk of malignant formation or* in vivo* differentiation and the release of cytokines, and finally to improve engraftment. These are the crucial issues which have to be addressed in future research to reach clinical utility and viability.

## Figures and Tables

**Box 1:**
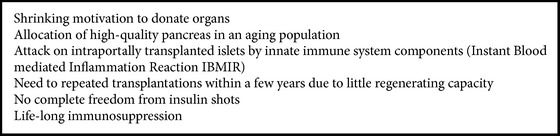
**Box 1: **Detriments of cadaveric human islet transplantation.

**Table 1:** Factors involved in beta-cell differentiation.

Compound	Role in beta-cell differentiation
High glucose	Increases beta-cell replication
Activin A	Promotes beta-cell regeneration and increases cell mass
N2 and B27 supplements	Act as serum supplements in serum-free medium
FGF2	Is important in early stage differentiation
EGF	Accelerates beta-cell proliferation and supports final stages of insulin expression
Beta-cellulin	Promotes beta-cell regeneration and increases cell mass
HGF	Induces beta-cell formation, especially in combination with activin A
RA	Induces endocrine and ductal differentiation and induces insulin-positive differentiation
GLP-1	Accelerates maturation of beta-cells towards glucose responsive insulin secretion
Exendin-4	Accelerates maturation of beta-cells towards glucose responsive insulin secretion
Pentagastrin	Expands beta-cell mass in combination with other factors

**Table 2:** Resultant expression of key pancreatic transcription factors, hormones, and improvement of diabetes *in vivo* after differentiation of MSCs via chemical compounds.

	Cell source	PDX-1	FoxA2/HNF3*β*	NGN-3	Pax-4	Pax-6	NeuroD	Isl-1	Glut-2	GCK	Insulin	Glucagon	Somatostatin	C-peptide	DTZ staining	GSIS *in vitro*	GSIS *in vivo*	Ameliorates diabetes
Dave et al. 2014 [52]	Adipose tissue	+	ND	ND	ND	+	ND	+	ND	ND	+	ND	ND	+	ND	+	Tx human	
Li et al. 2013 [53]		+	+	ND	ND	+	ND	ND	+	ND	+	+	Like basal	+	ND	+	ND	ND
Marappagounder et al. 2013 [51]		+	ND	+	+	ND	ND	+	ND	ND	+	ND	ND	ND	+	+	ND	ND
Mohamad Buang et al. 2012 [54]		ND	ND	ND	ND	ND	ND	ND	ND	ND	+	ND	ND	ND	+	+		
Chandra et al. 2011 [50]		+	+	+	+	+	+	+	+	ND	+	+	+	+	ND	+	+	+
Timper et al. 2006 [49]		+	ND	+	ND	Like basal	ND	+	ND	ND	+	+	+	+	ND	ND	ND	ND

Gabr et al. 2015 [56]	Bone marrow	+ (Tx)	ND	ND	ND	ND	ND	ND	+ (Tx)	+ (Tx)	+ (Tx)	+ (Tx)	+ (Tx)	+ (Tx)		ND	+ (Tx)	+ (Tx)
Czubak et al. 2014 [55]		ND	ND	ND	ND	ND	ND	ND	ND	ND	ND	ND	ND	+	+			
Jafarian et al. 2014 [57]		+	+	+	ND	ND	ND	ND	+	ND	+	+	+	+	+	+		
Marappagounder et al. 2013 [51]		+	ND	+	+	ND	ND	+	ND	ND	+	ND	ND	ND	+	+	ND	ND

ND = not determined, Tx after transplantation of MSC, and Tx (human) = recent clinical trial [61].

**Table 3:** Overview of studies addressing ectopic transduction of genes to promote beta-cell differentiation in BM-MSC and AD-MSC. Resultant expression of key pancreatic transcription factors, hormones, and improvement of diabetes *in vivo*.

	Cell source	PDX-1	FoxA2/HNF3*β*	NGN-3	Pax-4	Pax-6	NeuroD	Isl-1	Glut-2	GCK	Insulin	Glucagon	Somatostatin	C-peptide	DTZ staining	GSIS *in vitro*	GSIS *in vivo*	Ameliorates diabetes
	*Adipose tissue*																	
Bahrebar et al. 2015 [62]	LV-Pdx1	+	ND	+	ND	ND	ND	ND	ND	ND	+	ND	ND	ND	+	+	ND	ND
Lee et al. 2013 [63]	pAd-Pdx1-ZSGreen	+	+	ND	ND	ND	+	ND	+	+	+	ND	ND	+	ND	+	+	−
Boroujeni and Aleyasin 2013 [64]	LV-Pdx1	+	ND	+	ND	ND	ND	ND	+	ND	+	+	+	ND	ND	+	+	+
Lin et al. 2009 [65]	LV-Pdx1	+	ND	ND	ND	ND	+	ND	ND	ND	+	+	ND	ND	ND	+	ND	+

	*Bone marrow*																	
Jafarian et al. 2015 [66]	miR-375, anti-miR-9	+	+	+	ND	ND	ND	ND	+	ND	+	+	+	+	+	+	ND	ND
Allahverdi et al. 2015 [67]	LV-Pdx1	+	ND	ND	ND	ND	ND	ND	ND	ND	+	ND	ND	+	ND	+	ND	ND
Limbert et al. 2011 [68]	Pdx1-pDNA6/Ngn3-pcDNA3.1	+	ND	+	+	+	+	+	+	+	+	+	+	+	ND	−	ND	ND
Limbert et al. 2011 [68]	Pdx1-pDNA6	+	ND	−	+	+	+	+	+	+	+	+	+	+	ND	−	ND	ND
Limbert et al. 2011 [68]	Ngn3-pcDNA3.1	+	ND	+	+	+	+	+	+	+	+	+	+	+	ND	−	ND	ND
Karnieli et al. 2007 [69]	RV-Pdx1	+	ND	−	+	Like basal	−	Like basal	+	+	+	+	Like basal	+	ND	+	ND	+

## Common Abbreviations

AD-MSCs: Adipose tissue derived mesenchymal stem cells
ALCAM: Activated leukocyte cell adhesion molecule
AM-MSCs: Amniotic membrane and fluid mesenchymal stem cells
ANG: Angiogenin
APJ: Apelin receptor, a G-protein coupled receptor
ASCs: Adipose tissue derived stem cells
AT-MSCs: Adipose tissue derived stem cells
Bcl-2: Founding member of the b-cell lymphoma-2 regulator proteins and correspondent genes that regulate apoptosis
BM-MSCs: Bone marrow derived mesenchymal stem cells
Brachyury: Transcription factor within the T-box complex of genes highly expressed in inner cell mass of blastocyst, defining midline of a bilateral organism
CD: Cluster of differentiation
CITR: Collaborative Islet Transplant Registry
c-myc: V-myc avian myelocytomatosis viral oncogene homolog
Cxcr4: Gene encoding the CXC (cysteine-x-cysteine motif) chemokine receptor type 4
DMEM: Dulbecco's Modified Eagle Medium, LG = low glucose, and HG = high glucose
DPSCs: Dental pulp mesenchymal stem cells
DTZ: Dithizone, sulfur-containing organic compound forming complexes with metal cations, such as Zn, used to assess the purity of islet cell preparations
EGF: Epithelial growth factor
ESCs: Embryonic stem cells
FBS: Fetal bovine serum
FcR gamma: Fc receptor gamma
FGF: Fibroblast growth factor
FGF2: Fibroblast growth factor 2
FISH: Fluorescent DNA* in situ* hybridization
FoxA2: Gene encoding Forkhead box protein A2 transcription factor
GFR: Glomerular filtration rate
Glut2: Glucose transporter 2
Glp-1: Glucagon-like peptide-1
GM-CSF: Colony stimulating factor 2 (granulocyte-macrophage)
GK: Glucokinase
GMP: Good manufacturing practice
GPIIB: Glycoprotein IIB
GPIIIa: Glycoprotein IIIa
GPVI: Glycoprotein VI
GR1: Granulocyte marker 1
GTPs: Good tissue practices
HbA_1c_: Hemoglobin glycosylated
HBSS: Hank's Balanced Salt Solution
HGF: Hepatocyte growth factor
HIF-1: Hypoxia-inducible factor 1
HLA-DR: Human leukocyte antigen receptor D
HNF2: Hepatocyte nuclear factor 2
IBMIR: Instant blood mediated immune reaction
ICAM-1: Intercellular adhesion molecule 1
IFN-gamma: Interferon gamma
IPCs: Insulin-producing cells
iPSCs: Induced pluripotent stem cells
IL: Interleukin
ISCT: International Society for Cellular Therapy
Isl1: Insulin gene enhancer protein 1, a protein encoded by the correspondent gene
MafA: V-maf musculoaponeurotic fibrosarcoma oncogene homolog A, a protein encoded by the MafA gene
MCP-1: Monocyte chemotactic protein 1
Mixl1: Mix paired-like homeobox gene encoding transcription factor
MMP: Metalloprotease
MOI: Multiplicity of infection
MSCs: Mesenchymal stem cells
MSCA-1: Mesenchymal stem cell antigen-1
NANOG: (Old Irish Tir na nOg = land of youth) gene encoding a transcription factor regulating self-renewal in stem cells
NCV: Nerve conduction velocity
NK cells: Natural killer cells
NeuroD1: Neurogenic Differentiation 1 gene encoding basic helix loop helix transcription factor of the NeuroD family
Ngn3: Neurogenin gene encoding class A basic helix loop helix transcription factor
Nkx2.2 NK2: Homeobox 2 transcription factor encoded by correspondent gene also named TTF1
NO: Nitric oxide
OCT4: Octamer binding transcription factor 4
P-MSC: Pancreas
PL-MSC: Placenta
PAF: Platelet activating factor
Pax6: Gene encoding the transcription factor Paired box protein 6
PDGF: Platelet derived growth factor
PDX-1: Pancreatic and duodenal homeobox 1, synonymous with insulin promoter factor 1
PP: Pancreatic progenitor
RA: Retinoic acid
SCF: Stem cell factor, a cytokine produced by fibroblasts and endothelial cells, in its soluble form binding to c-KIT (CD117)
SOX2: Sex-determining region-related High-Mobility-Group-Box gene 2
STRO-1: Stromal cell derived factor 1
STRO-3: Stromal cell derived factor 3
STZ: Streptozotocin, chemical for induction of experimental diabetes in rodents
STZ mice: Streptozotocin-induced diabetes in mice
T1DM: Type 1 diabetes mellitus
TCDAb: T-cell depleting antibody
TGF: Tissue growth factor
TGF beta: Tissue growth factor beta
Thy1: Thymocyte differentiation antigen 1
TNF-alpha: Tumor necrosis factor alpha
TNF-alpha-i: Tumor necrosis factor alpha inhibitor
TXA: Thromboxane
UC-MSCs: Umbilical cord matrix mesenchymal stem cells
UCB-MSCs: Umbilical cord blood mesenchymal stem cells
VCAM: Vascular cell adhesion molecule-1
VEGF: Vascular endothelial growth factor
VLA-4: Integrin alpha4beta1
Wnt: Wingless Int-1 characterizing a group of proteins passing signals from outside to inside of the cell membrane
WT-MSCs: Wharton's jelly mesenchymal stem cells.

## References

[1] Shapiro A. M. J., Lakey J. R. T., Ryan E. A. (2000). Islet transplantation in seven patients with type 1 diabetes mellitus using a glucocorticoid-free immunosuppressive regimen. *The New England Journal of Medicine*.

[2] Bretzel R. G., Jahr H., Eckhard M., Martin I., Winter D., Brendel M. D. (2007). Islet cell transplantation today. *Langenbeck's Archives of Surgery*.

[3] McCall M., Shapiro A. M. J. (2014). Islet cell transplantation. *Seminars in Pediatric Surgery*.

[4] The Diabetes Control and Complications Trial Research Group (1997). Hypoglycemia in the diabetes control and complications trial. *Diabetes*.

[5] Ryan E. A., Lakey J. R. T., Paty B. W. (2002). Successful islet transplantation: continued insulin reserve provides long-term glycemic control. *Diabetes*.

[6] Ryan E. A., Lakey J. R. T., Rajotte R. V. (2001). Clinical outcomes and insulin secretion after islet transplantation with the Edmonton protocol. *Diabetes*.

[7] Shapiro A. M. J., Ricordi C., Hering B. J. (2006). International trial of the Edmonton protocol for islet transplantation. *The New England Journal of Medicine*.

[8] Hirshberg B., Rother K. I., Digon B. J. (2003). Benefits and risks of solitary islet transplantation for type 1 diabetes using steroid-sparing immunosuppression. *Diabetes Care*.

[9] Kenmochi T., Asano T., Maruyama M. (2009). Clinical islet transplantation in Japan. *Journal of Hepato-Biliary-Pancreatic Surgery*.

[10] Ryan E. A., Shandro T., Green K. (2004). Assessment of the severity of hypoglycemia and glycemic lability in type 1 diabetic subjects undergoing islet transplantation. *Diabetes*.

[11] Ryan E. A., Paty B. W., Senior P. A. (2005). Five-year follow-up after clinical islet transplantation. *Diabetes*.

[12] Hering B. J., Kandaswamy R., Ansite J. D. (2005). Single-donor, marginal-dose islet transplantation in patients with type 1 diabetes. *The Journal of the American Medical Association*.

[13] Bellin M. D., Kandaswamy R., Parkey J. (2008). Prolonged insulin independence after islet allotransplants in recipients with type 1 diabetes. *American Journal of Transplantation*.

[14] Bellin M. D., Barton F. B., Heitman A. (2012). Potent induction immunotherapy promotes long-term insulin independence after islet transplantation in type 1 diabetes. *The American Journal of Transplantation*.

[15] Froud T., Ricordi C., Baidal D. A. (2005). Islet transplantation in type 1 diabetes mellitus using cultured islets and steroid-free immunosuppression: Miami experience. *American Journal of Transplantation*.

[16] O'Connell P. J., Holmes-Walker D. J., Goodman D. (2013). Multicenter Australian trial of islet transplantation: improving accessibility and outcomes. *American Journal of Transplantation*.

[17] Qi M., Kinzer K., Danielson K. K. (2014). Five-year follow-up of patients with type 1 diabetes transplanted with allogeneic islets: the UIC experience. *Acta Diabetologica*.

[18] Turgeon N. A., Avila J. G., Cano J. A. (2010). Experience with a novel efalizumab-based immunosuppressive regimen to facilitate single donor islet cell transplantation. *American Journal of Transplantation*.

[19] Ludwig B., Reichel A., Kruppa A. (2015). Islet transplantation at the Dresden diabetes center: five years' experience. *Hormone and Metabolic Research*.

[20] Barton F. B., Rickels M. R., Alejandro R. (2012). Improvement in outcomes of clinical islet transplantation: 1999-2010. *Diabetes Care*.

[21] Rickels M. R., Liu C., Shlansky-Goldberg R. D. (2013). Improvement in *β*-cell secretory capacity after human islet transplantation according to the CIT07 protocol. *Diabetes*.

[22] Vantyghem M.-C., Raverdy V., Balavoine A.-S. (2078). Continuous glucose monitoring after islet transplantation in type 1 diabetes: an excellent graft function (*β*-score greater than 7) is required to abrogate hyperglycemia, whereas a minimal function is necessary to suppress severe hypoglycemia (*β*-score greater than 3). *The Journal of Clinical Endocrinology & Metabolism*.

[23] Ang M., Meyer C., Brendel M. D., Bretzel R. G., Linn T. (2014). Magnitude and mechanisms of glucose counterregulation following islet transplantation in patients with type 1 diabetes suffering from severe hypoglycaemic episodes. *Diabetologia*.

[24] Rickels M. R., Schutta M. H., Mueller R. (2007). Glycemic thresholds for activation of counterregulatory hormone and symptom responses in islet transplant recipients. *Journal of Clinical Endocrinology and Metabolism*.

[25] Rickels M. R., Schutta M. H., Mueller R. (2005). Islet cell hormonal responses to hypoglycemia after human islet transplantation for type 1 diabetes. *Diabetes*.

[26] Leitão C. B., Tharavanij T., Cure P. (2008). Restoration of hypoglycemia awareness after islet transplantation. *Diabetes Care*.

[27] Rickels M. R., Kong S. M., Fuller C. (2013). Improvement in insulin sensitivity after human islet transplantation for type 1 diabetes. *Journal of Clinical Endocrinology and Metabolism*.

[28] Toso C., Shapiro A. M. J., Bowker S. (2007). Quality of life after islet transplant: impact of the number of islet infusions and metabolic outcome. *Transplantation*.

[29] Fullerton B., Jeitler K., Seitz M., Horvath K., Berghold A., Siebenhofer A. (2014). Intensive glucose control versus conventional glucose control for type 1 diabetes mellitus. *The Cochrane Database of Systematic Reviews*.

[30] Diabetes Control and Complications Trial Research Group (1993). The effect of intensive treatment of diabetes on the development and progression of long-term complications in insulin-dependent diabetes mellitus. *The New England Journal of Medicine*.

[31] Wang P. H., Lau J., Chalmers T. C. (1993). Meta-analysis of effects of intensive blood-glucose control on late complications of type I diabetes. *The Lancet*.

[32] Fiorina P., Folli F., Zerbini G. (2003). Islet transplantation is associated with improvement of renal function among uremic patients with type I diabetes mellitus and kidney transplants. *Journal of the American Society of Nephrology*.

[33] Fiorina P., Venturini M., Folli F. (2005). Natural history of kidney graft survival, hypertrophy, and vascular function in end-stage renal disease type 1 diabetic kidney-transplanted patients: beneficial impact of pancreas and successful islet cotransplantation. *Diabetes Care*.

[34] Andres A., Toso C., Morel P. (2005). Impact of a sirolimus/tacrolimus-based immunosuppressive regimen on kidney function after islet transplantation. *Transplantation Proceedings*.

[35] Maffi P., Bertuzzi F., De Taddeo F. (2007). Kidney function after islet transplant alone in type 1 diabetes: impact of immunosuppressive therapy on progression of diabetic nephropathy. *Diabetes Care*.

[36] Senior P. A., Zeman M., Paty B. W., Ryan E. A., Shapiro A. M. J. (2007). Changes in renal function after clinical islet transplantation: four-year observational study. *American Journal of Transplantation*.

[37] Fung M. A., Warnock G. L., Ao Z. (2007). The effect of medical therapy and islet cell transplantation on diabetic nephropathy: an interim report. *Transplantation*.

[38] Warnock G. L., Thompson D. M., Meloche R. M. (2008). A multi-year analysis of islet transplantation compared with intensive medical therapy on progression of complications in type 1 diabetes. *Transplantation*.

[39] Thompson D. M., Meloche M., Ao Z. (2011). Reduced progression of diabetic microvascular complications with islet cell transplantation compared with intensive medical therapy. *Transplantation*.

[40] Lee T. C., Barshes N. R., O'Mahony C. A. (2005). The effect of pancreatic islet transplantation on progression of diabetic retinopathy and neuropathy. *Transplantation Proceedings*.

[41] Venturini M., Fiorina P., Maffi P. (2006). Early increase of retinal arterial and venous blood flow velocities at color doppler imaging in brittle type 1 diabetes after islet transplant alone. *Transplantation*.

[42] Thompson D. M., Begg I. S., Harris C. (2008). Reduced progression of diabetic retinopathy after islet cell transplantation compared with intensive medical therapy. *Transplantation*.

[43] Del Carro U., Fiorina P., Amadio S. (2007). Evaluation of polyneuropathy markers in type 1 diabetic kidney transplant patients and effects of islet transplantation: neurophysiological and skin biopsy longitudinal analysis. *Diabetes Care*.

[44] Vantyghem M.-C., Quintin D., Caiazzo R. (2014). Improvement of electrophysiological neuropathy after islet transplantation for type 1 diabetes: a 5-year prospective study. *Diabetes Care*.

[45] Fiorina P., Folli F., Bertuzzi F. (2003). Long-term beneficial effect of islet transplantation on diabetic macro-/microangiopathy in type 1 diabetic kidney-transplanted patients. *Diabetes Care*.

[46] Fiorina P., Folli F., Maffi P. (2003). Islet transplantation improves vascular diabetic complications in patients with diabetes who underwent kidney transplantation: a comparison between kidney-pancreas and kidney-alone transplantation. *Transplantation*.

[47] Fiorina P., Gremizzi C., Maffi P. (2005). Islet transplantation is associated with an improvement of cardiovascular function in type 1 diabetic kidney transplant patients. *Diabetes Care*.

[48] D'Addio F., Maffi P., Vezzulli P. (2014). Islet transplantation stabilizes hemostatic abnormalities and cerebral metabolism in individuals with type 1 diabetes. *Diabetes Care*.

[49] Timper K., Seboek D., Eberhardt M. (2006). Human adipose tissue-derived mesenchymal stem cells differentiate into insulin, somatostatin, and glucagon expressing cells. *Biochemical and Biophysical Research Communications*.

[50] Chandra V., Swetha G., Muthyala S. (2011). Islet-like cell aggregates generated from human adipose tissue derived stem cells ameliorate experimental diabetes in mice. *PLoS ONE*.

[51] Marappagounder D., Somasundaram I., Dorairaj S., Sankaran R. J. (2013). Differentiation of mesenchymal stem cells derived from human bone marrow and subcutaneous adipose tissue into pancreatic islet-like clusters in vitro. *Cellular and Molecular Biology Letters*.

[52] Dave S. D., Vanikar A. V., Trivedi H. L. (2014). In-vitro generation of human adipose tissue derived insulin secreting cells: up-regulation of Pax-6, Ipf-1 and Isl-1. *Cytotechnology*.

[53] Li J., Zhu L., Qu X. (2013). Stepwise differentiation of human adipose-derived mesenchymal stem cells toward definitive endoderm and pancreatic progenitor cells by mimicking pancreatic development in vivo. *Stem Cells and Development*.

[54] Mohamad Buang M. L., Seng H. K., Chung L. H., Saim A. B., Idrus R. B. H. (2012). In vitro generation of functional insulin-producing cells from lipoaspirated human adipose tissue-derived stem cells. *Archives of Medical Research*.

[55] Czubak P., Bojarska-Junak A., Tabarkiewicz J., Putowski L. (2014). A modified method of insulin producing cells' generation from bone marrow-derived mesenchymal stem cells. *Journal of Diabetes Research*.

[56] Gabr M. M., Zakaria M. M., Refaie A. F. (2015). Differentiation of human bone marrow-derived mesenchymal stem cells into insulin-producing cells: evidence for further maturation in vivo. *BioMed Research International*.

[57] Jafarian A., Taghikhani M., Abroun S., Pourpak Z., Allahverdi A., Soleimani M. (2014). Generation of high-yield insulin producing cells from human bone marrow mesenchymal stem cells. *Molecular Biology Reports*.

[58] Bennet W., Sundberg B., Groth C.-G. (1999). Incompatibility between human blood and isolated islets of langerhans: a finding with implications for clinical intraportal islet transplantation?. *Diabetes*.

[59] Bennet W., Groth C.-G., Larsson R., Nilsson B., Korsgren O. (2000). Isolated human islets trigger an instant blood mediated inflammatory reaction: implications for intraportal islet transplantation as a treatment for patients with type 1 diabetes. *Upsala Journal of Medical Sciences*.

[60] Lai Y., Chen C., Linn T. (2009). Innate immunity and heat shock response in islet transplantation. *Clinical and Experimental Immunology*.

[61] Thakkar U. G., Trivedi H. L., Vanikar A. V., Dave S. D. (2015). Insulin-secreting adipose-derived mesenchymal stromal cells with bone marrow-derived hematopoietic stem cells from autologous and allogenic sources for type 1 diabetes mellitus. *Cytotherapy*.

[62] Bahrebar M., Soleimani M., Karimi M. H., Vahdati A., Yaghobi R. (2015). Generation of islet-like cell aggregates from human adipose tissue-derived stem cells by lentiviral overexpression of PDX-1. *International Journal of Organ Transplantation Medicine*.

[63] Lee J., Kim S. C., Kim S. J. (2013). Differentiation of human adipose tissue-derived stem cells into aggregates of insulin-producing cells through the overexpression of pancreatic and duodenal homeobox gene-1. *Cell Transplantation*.

[64] Boroujeni Z. N., Aleyasin A. (2013). Insulin producing cells established using non-integrated lentiviral vector harboring *PDX1* gene. *World Journal of Stem Cells*.

[65] Lin G., Wang G., Liu G. (2009). Treatment of type 1 diabetes with adipose tissue-derived stem cells expressing pancreatic duodenal homeobox 1. *Stem Cells and Development*.

[66] Jafarian A., Taghikani M., Abroun S. (2015). The generation of insulin producing cells from human mesenchymal stem cells by MiR-375 and anti-MiR-9. *PLoS ONE*.

[67] Allahverdi A., Abroun S., Jafarian A., Soleimani M., Taghikhani M., Eskandari F. (2015). Differentiation of human mesenchymal stem cells into insulin producing cells by using a lentiviral vector carrying PDX1. *Cell Journal*.

[68] Limbert C., Pth G., Ebert R. (2011). PDX1- and NGN3-mediated in vitro reprogramming of human bone marrow-derived mesenchymal stromal cells into pancreatic endocrine lineages. *Cytotherapy*.

[69] Karnieli O., Izhar-Prato Y., Bulvik S., Efrat S. (2007). Generation of insulin-producing cells from human bone marrow mesenchymal stem cells by genetic manipulation. *STEM CELLS*.

[70] Maloney J. P., Silliman C. C., Ambruso D. R., Wang J., Tuder R. M., Voelkel N. F. (1998). In vitro release of vascular endothelial growth factor during platelet aggregation. *The American Journal of Physiology—Heart and Circulatory Physiology*.

[71] Nieswandt B., Bergmeier W., Eckly A. (2001). Evidence for cross-talk between glycoprotein VI and Gi-coupled receptors during collagen-induced platelet aggregation. *Blood*.

[72] Nieswandt B., Pleines I., Bender M. (2011). Platelet adhesion and activation mechanisms in arterial thrombosis and ischaemic stroke. *Journal of Thrombosis and Haemostasis*.

[73] Nieswandt B., Brakebusch C., Bergmeier W. (2001). Glycoprotein VI but not *α*2*β*1 integrin is essential for platelet interaction with collagen. *The EMBO Journal*.

[74] Nieswandt B., Schulte V., Bergmeier W. (2001). Long-term antithrombotic protection by in vivo depletion of platelet glycoprotein VI in mice. *The Journal of Experimental Medicine*.

[75] Linn T., Schmitz J., Hauck-Schmalenberger I. (2006). Ischaemia is linked to inflammation and induction of angiogenesis in pancreatic islets. *Clinical and Experimental Immunology*.

[76] Barshes N. R., Wyllie S., Goss J. A. (2005). Inflammation-mediated dysfunction and apoptosis in pancreatic islet transplantation: implications for intrahepatic grafts. *Journal of Leukocyte Biology*.

[77] Emamaullee J. A., Shapiro A. M. J. (2007). Factors influencing the loss of *β*-cell mass in islet transplantation. *Cell Transplantation*.

[78] Yasunami Y., Kojo S., Kitamura H. (2005). V*α*14 NKT cell-triggered IFN-*α* production by Gr-1^+^CD11b^+^ cells mediates early graft loss of syngeneic transplanted islets. *The Journal of Experimental Medicine*.

[79] Brandhorst H., Brandhorst D., Hering B. J., Bretzel R. G. (1999). Significant progress in porcine islet mass isolation utilizing liberase HI for enzymatic low-temperature pancreas digestion. *Transplantation*.

[80] Shapiro A. M. J., Hao E., Rajotte R. V., Kneteman N. M. (1996). High yield of rodent islets with intraductal collagenase and stationary digestion—a comparison with standard technique. *Cell Transplantation*.

[81] Brandhorst H., Brandhorst D., Brendel M. D., Hering B. J., Bretzel R. G. (1998). Assessment of intracellular insulin content during all steps of human islet isolation procedure. *Cell Transplantation*.

[82] Piemonti L., Pileggi A. (2013). 25 Years of the Ricordi automated method for islet isolation. *CellR4*.

[83] Brandhorst H., Raemsch-Guenther N., Raemsch C. (2008). The ratio between collagenase class I and class II influences the efficient islet release from the rat pancreas. *Transplantation*.

[84] Carter J. D., Dula S. B., Corbin K. L., Wu R., Nunemaker C. S. (2009). A practical guide to rodent islet isolation and assessment. *Biological Procedures Online*.

[85] Gaber A. O., Fraga D. (2004). Advances in long-term islet culture: the Memphis experience. *Cell Biochemistry and Biophysics*.

[86] Ramnath R. D., Maillard E., Jones K. (2015). In vitro assessment of human islet vulnerability to instant blood-mediated inflammatory reaction (IBMIR) and its use to demonstrate a beneficial effect of tissue culture. *Cell Transplantation*.

[87] Carlsson P.-O., Schwarcz E., Korsgren O., Le Blanc K. (2015). Preserved *β*-cell function in type 1 diabetes by mesenchymal stromal cells. *Diabetes*.

[88] Hu J., Yu X., Wang Z. (2013). Long term effects of the implantation of Wharton's jelly-derived mesenchymal stem cells from the umbilical cord for newly-onset type 1 diabetes mellitus. *Endocrine Journal*.

[89] Friedenstein A. J., Petrakova K. V., Kurolesova A. I., Frolova G. P. (1968). Heterotopic of bone marrow. Analysis of precursor cells for osteogenic and hematopoietic tissues. *Transplantation*.

[90] Owen M. (1988). Marrow stromal stem cells. *Journal of Cell Science*.

[91] Owen M., Friedenstein A. J. (1988). Stromal stem cells: marrow-derived osteogenic precursors. *Ciba Foundation Symposium*.

[92] Caplan A. I. (1991). Mesenchymal stem cells. *Journal of Orthopaedic Research*.

[93] Williams J. T., Southerland S. S., Souza J., Calcutt A. F., Cartledge R. G. (1999). Cells isolated from adult human skeletal muscle capable of differentiating into multiple mesodermal phenotypes. *American Surgeon*.

[94] Bartsch G., Yoo J. J., De Coppi P. (2005). Propagation, expansion, and multilineage differentiation of human somatic stem cells from dermal progenitors. *Stem Cells and Development*.

[95] Riekstina U., Muceniece R., Cakstina I., Muiznieks I., Ancans J. (2008). Characterization of human skin-derived mesenchymal stem cell proliferation rate in different growth conditions. *Cytotechnology*.

[96] Zuk P. A., Zhu M., Mizuno H. (2001). Multilineage cells from human adipose tissue: implications for cell-based therapies. *Tissue Engineering*.

[97] Seeberger K. L., Dufour J. M., James Shapiro A. M., Lakey J. R. T., Rajotte R. V., Korbutt G. S. (2006). Expansion of mesenchymal stem cells from human pancreatic ductal epithelium. *Laboratory Investigation*.

[98] Gronthos S., Mankani M., Brahim J., Robey P. G., Shi S. (2000). Postnatal human dental pulp stem cells (DPSCs) in vitro and in vivo. *Proceedings of the National Academy of Sciences of the United States of America*.

[99] Rotter N., Oder J., Schlenke P. (2008). Isolation and characterization of adult stem cells from human salivary glands. *Stem Cells and Development*.

[100] Schüring A. N., Schulte N., Kelsch R., Röpke A., Kiesel L., Götte M. (2011). Characterization of endometrial mesenchymal stem-like cells obtained by endometrial biopsy during routine diagnostics. *Fertility and Sterility*.

[101] Fukuchi Y., Nakajima H., Sugiyama D., Hirose I., Kitamura T., Tsuji K. (2004). Human placenta-derived cells have mesenchymal stem/progenitor cell potential. *STEM CELLS*.

[102] Hoyes A. D. (1975). Structure and function of the amnion. *Obstetrics and Gynecology Annual*.

[103] Macek M., Hurych J., Rezacova D. (1973). Collagen synthesis in long-term cultures of amniotic fluid. *Ceskoslovenská Pediatrie*.

[104] Macek M., Hurych J., Rezacova D. (1973). Letter: collagen synthesis in long term amniotic fluid cell cultures. *Nature*.

[105] Pappa K. I., Anagnou N. P. (2009). Novel sources of fetal stem cells: where do they fit on the developmental continuum?. *Regenerative Medicine*.

[106] Erices A., Conget P., Minguell J. J. (2000). Mesenchymal progenitor cells in human umbilical cord blood. *British Journal of Haematology*.

[107] Romanov Y. A., Svintsitskaya V. A., Smirnov V. N. (2003). Searching for alternative sources of postnatal human mesenchymal stem cells: candidate MSC-like cells from umbilical cord. *STEM CELLS*.

[108] Mitchell K. E., Weiss M. L., Mitchell B. M. (2003). Matrix cells from Wharton's jelly form neurons and glia. *Stem Cells*.

[109] Pittenger M. F., Mackay A. M., Beck S. C. (1999). Multilineage potential of adult human mesenchymal stem cells. *Science*.

[110] Sanchez-Ramos J., Song S., Cardozo-Pelaez F. (2000). Adult bone marrow stromal cells differentiate into neural cells in vitro. *Experimental Neurology*.

[111] Wagner W., Wein F., Seckinger A. (2005). Comparative characteristics of mesenchymal stem cells from human bone marrow, adipose tissue, and umbilical cord blood. *Experimental Hematology*.

[112] Winter A., Breit S., Parsch D. (2003). Cartilage-like gene expression in differentiated human stem cell spheroids: a comparison of bone marrow-derived and adipose tissue-derived stromal cells. *Arthritis and Rheumatism*.

[113] Planat-Benard V., Silvestre J.-S., Cousin B. (2004). Plasticity of human adipose lineage cells toward endothelial cells: physiological and therapeutic perspectives. *Circulation*.

[114] Safford K. M., Hicok K. C., Safford S. D. (2002). Neurogenic differentiation of murine and human adipose-derived stromal cells. *Biochemical and Biophysical Research Communications*.

[115] Stock P., Brückner S., Winkler S., Dollinger M. M., Christ B. (2014). Human bone marrow mesenchymal stem cell-derived hepatocytes improve the mouse liver after acute acetaminophen intoxication by preventing progress of injury. *International Journal of Molecular Sciences*.

[116] Wilkins A., Kemp K., Ginty M., Hares K., Mallam E., Scolding N. (2009). Human bone marrow-derived mesenchymal stem cells secrete brain-derived neurotrophic factor which promotes neuronal survival in vitro. *Stem Cell Research*.

[117] Orlic D., Kajstura J., Chimenti S. (2001). Bone marrow cells regenerate infarcted myocardium. *Nature*.

[118] Horwitz E. M., Prockop D. J., Fitzpatrick L. A. (1999). Transplantability and therapeutic effects of bone marrow-derived mesenchymal cells in children with osteogenesis imperfecta. *Nature Medicine*.

[119] Morigi M., Imberti B., Zoja C. (2004). Mesenchymal stem cells are renotropic, helping to repair the kidney and improve function in acute renal failure. *Journal of the American Society of Nephrology*.

[120] Schwartz R. E., Reyes M., Koodie L. (2002). Multipotent adult progenitor cells from bone marrow differentiate into functional hepatocyte-like cells. *The Journal of Clinical Investigation*.

[121] Dominici M., Le Blanc K., Mueller I. (2006). Minimal criteria for defining multipotent mesenchymal stromal cells. The International Society for Cellular Therapy position statement. *Cytotherapy*.

[122] Horwitz E. M., Le Blanc K., Dominici M. (2005). Clarification of the nomenclature for MSC: The International Society for Cellular Therapy position statement. *Cytotherapy*.

[123] Sauer H., Sharifpanah F., Hatry M. (2011). NOS inhibition synchronizes calcium oscillations in human adipose tissue-derived mesenchymal stem cells by increasing gap-junctional coupling. *Journal of Cellular Physiology*.

[124] Simmons P. J., Torok-Storb B. (1991). Identification of stromal cell precursors in human bone marrow by a novel monoclonal antibody, STRO-1. *Blood*.

[125] Gronthos S., Fitter S., Diamond P., Simmons P. J., Itescu S., Zannettino A. C. W. (2007). A novel monoclonal antibody (STRO-3) identifies an isoform of tissue nonspecific alkaline phosphatase expressed by multipotent bone marrow stromal stem cells. *Stem Cells and Development*.

[126] Battula V. L., Treml S., Bareiss P. M. (2009). Isolation of functionally distinct mesenchymal stem cell subsets using antibodies against CD56, CD271, and mesenchymal stem cell antigen-1. *Haematologica*.

[127] Quirici N., Soligo D., Bossolasco P., Servida F., Lumini C., Deliliers G. L. (2002). Isolation of bone marrow mesenchymal stem cells by anti-nerve growth factor receptor antibodies. *Experimental Hematology*.

[128] Aye M. T., Hashemi S., Leclair B. (1992). Expression of stem cell factor and c-kit mRNA in cultured endothelial cells, monocytes and cloned human bone marrow stromal cells (CFU-RF). *Experimental Hematology*.

[129] Strem B. M., Hicok K. C., Zhu M. (2005). Multipotential differentiation of adipose tissue-derived stem cells. *Keio Journal of Medicine*.

[130] De Ugarte D. A., Alfonso Z., Zuk P. A. (2003). Differential expression of stem cell mobilization-associated molecules on multi-lineage cells from adipose tissue and bone marrow. *Immunology Letters*.

[131] De Ugarte D. A., Morizono K., Elbarbary A. (2003). Comparison of multi-lineage cells from human adipose tissue and bone marrow. *Cells Tissues Organs*.

[132] Simmons P. J., Masinovsky B., Longenecker B. M., Berenson R., Torok-Storb B., Gallatin W. M. (1992). Vascular cell adhesion molecule-1 expressed by bone marrow stromal cells mediates the binding of hematopoietic progenitor cells. *Blood*.

[133] Südhoff T., Söhngen D. (2002). Circulating endothelial adhesion molecules (sE-selectin, sVCAM-1 and sICAM-1) during rHUG-CSF-stimulated stem cell mobilization. *Journal of Hematotherapy and Stem Cell Research*.

[134] Auquier P., Macquart-Moulin G., Moatti J. (1995). Comparison of anxiety, pain and discomfort in two procedures of hematopoietic stem cell collection: leukacytapheresis and bone marrow harvest. *Bone Marrow Transplantation*.

[135] Dahl J.-A., Duggal S., Coulston N. (2008). Genetic and epigenetic instability of human bone marrow mesenchymal stem cells expanded in autologous seum or fatal bovine serum. *International Journal of Developmental Biology*.

[136] Meza-Zepeda L. A., Noer A., Dahl J. A., Micci F., Myklebost O., Collas P. (2008). High-resolution analysis of genetic stability of human adipose tissue stem cells cultured to senescence. *Journal of Cellular and Molecular Medicine*.

[137] Hess D., Li L., Martin M. (2003). Bone marrow–derived stem cells initiate pancreatic regeneration. *Nature Biotechnology*.

[138] Lee R. H., Seo M. J., Reger R. L. (2006). Multipotent stromal cells from human marrow home to and promote repair of pancreatic islets and renal glomeruli in diabetic NOD/scid mice. *Proceedings of the National Academy of Sciences of the United States of America*.

[139] Borg D. J., Weigelt M., Wilhelm C. (2014). Mesenchymal stromal cells improve transplanted islet survival and islet function in a syngeneic mouse model. *Diabetologia*.

[140] Aggarwal S., Pittenger M. F. (2005). Human mesenchymal stem cells modulate allogeneic immune cell responses. *Blood*.

[141] Ding Y., Xu D., Feng G., Bushell A., Muschel R. J., Wood K. J. (2009). Mesenchymal stem cells prevent the rejection of fully allogenic islet grafts by the immunosuppressive activity of matrix metalloproteinase-2 and -9. *Diabetes*.

[142] Ito T., Itakura S., Todorov I. (2010). Mesenchymal stem cell and islet co-transplantation promotes graft revascularization and function. *Transplantation*.

[143] Sordi V., Melzi R., Mercalli A. (2010). Mesenchymal cells appearing in pancreatic tissue culture are bone marrow-derived stem cells with the capacity to improve transplanted islet function. *Stem Cells*.

[144] Wilkinson D. G., Bhatt S., Herrmann B. G. (1990). Expression pattern of the mouse T gene and its role in mesoderm formation. *Nature*.

[145] Pearce J. J. H., Evans M. J. (1999). Mml, a mouse Mix-like gene expressed in the primitive streak. *Mechanisms of Development*.

[146] Ang S.-L., Wierda A., Wong D. (1993). The formation and maintenance of the definitive endoderm lineage in the mouse: involvement of HNF3/forkhead proteins. *Development*.

[147] Payne C., King J., Hay D. (2011). The role of activin/nodal and Wnt signaling in endoderm formation. *Vitamins and Hormones*.

[148] Kim S. K., Hebrok M. (2001). Intercellular signals regulating pancreas development and function. *Genes and Development*.

[149] Guney M. A., Gannon M. (2009). Pancreas cell fate. *Birth Defects Research Part C—Embryo Today: Reviews*.

[150] Jonsson J., Carlsson L., Edlund T., Edlund H. (1994). Insulin-promoter-factor 1 is required for pancreas development in mice. *Nature*.

[151] Gittes G. K. (2009). Developmental biology of the pancreas: a comprehensive review. *Developmental Biology*.

[152] Pictet R. L., Clark W. R., Williams R. H., Rutter W. J. (1972). An ultrastructural analysis of the developing embryonic pancreas. *Developmental Biology*.

[153] Furukawa M., Eto Y., Kojima I. (1995). Expression of immunoreactive activin A in fetal rat pancreas. *Endocrine Journal*.

[154] Apelqvist Å., Li H., Sommer L. (1999). Notch signalling controls pancreatic cell differentiation. *Nature*.

[155] Herrera P. L. (2000). Adult insulin- and glucagon-producing cells differentiate from two independent cell lineages. *Development*.

[156] Oliver-Krasinski J. M., Stoffers D. A. (2008). On the origin of the *β* cell. *Genes and Development*.

[157] Gradwohl G., Gradwohl G., Dierich A. (2000). Neurogenin3 is required for the development of the four endocrine cell lineages of the pancreas. *Proceedings of the National Academy of Sciences of the United States of America*.

[158] Schwitzgebel V. M., Scheel D. W., Conners J. R. (2000). Expression of neurogenin3 reveals an islet cell precursor population in the pancreas. *Development*.

[159] Wilson M. E., Scheel D., German M. S. (2003). Gene expression cascades in pancreatic development. *Mechanisms of Development*.

[160] Kim T., Gondré-Lewis M. C., Arnaoutova I., Loh Y. P. (2006). Dense-core secretory granule biogenesis. *Physiology*.

[161] Georgia S., Bhushan A. (2004). *β* cell replication is the primary mechanism for maintaining postnatal *β* cell mass. *Journal of Clinical Investigation*.

[162] Artner I., Blanchi B., Raum J. C. (2007). MafB is required for islet *β* cell maturation. *Proceedings of the National Academy of Sciences of the United States of America*.

[163] Aguayo-Mazzucato C., Koh A., El Khattabi I. (2011). Mafa expression enhances glucose-responsive insulin secretion in neonatal rat beta cells. *Diabetologia*.

[164] Goodyer W. R., Gu X., Liu Y., Bottino R., Crabtree G. R., Kim S. K. (2012). Neonatal *β* cell development in mice and humans is regulated by calcineurin/NFAT. *Developmental Cell*.

[165] Márquez-Aguirre A. L., Canales-Aguirre A. A., Padilla-Camberos E., Esquivel-Solis H., Díaz-Martínez N. E. (2015). Development of the endocrine pancreas and novel strategies for *β*-cell mass restoration and diabetes therapy. *Brazilian Journal of Medical and Biological Research*.

[166] Tang D. Q., Wang Q., Burkhardt B. R., Litherland S. A., Atkinson M. A., Yang L. J. (2012). In vitro generation of functional insulin-producing cells from human bone marrow-derived stem cells, but long-term culture running risk of malignant transformation. *American Journal of Stem Cells*.

[167] Tosh D., Slack J. M. W. (2002). How cells change their phenotype. *Nature Reviews Molecular Cell Biology*.

[168] Moriscot C., De Fraipont F., Richard M.-J. (2005). Human bone marrow mesenchymal stem cells can express insulin and key transcription factors of the endocrine pancreas developmental pathway upon genetic and/or microenvironmental manipulation in vitro. *STEM CELLS*.

[169] Dor Y., Brown J., Martinez O. I., Melton D. A. (2004). Adult pancreatic *β*-cells are formed by self-duplication rather than stem-cell differentiation. *Nature*.

[170] Kato T., Shimano H., Yamamoto T. (2006). Granuphilin is activated by SREBP-1c and involved in impaired insulin secretion in diabetic mice. *Cell Metabolism*.

[171] Kloosterman W. P., Lagendijk A. K., Ketting R. F., Moulton J. D., Plasterk R. H. A. (2007). Targeted inhibition of miRNA maturation with morpholinos reveals a role for miR-375 in pancreatic islet development. *PLoS Biology*.

[172] Plaisance V., Abderrahmani A., Perret-Menoud V., Jacquemin P., Lemaigre F., Regazzi R. (2006). MicroRNA-9 controls the expression of Granuphilin/Slp4 and the secretory response of insulin-producing cells. *The Journal of Biological Chemistry*.

[173] Poy M. N., Eliasson L., Krutzfeldt J. (2004). A pancreatic islet-specific microRNA regulates insulin secretion. *Nature*.

[174] Tang Y., Cai B., Yuan F. (2014). Melatonin pretreatment improves the survival and function of transplanted mesenchymal stem cells after focal cerebral ischemia. *Cell Transplantation*.

[175] Bhonde R. R., Sheshadri P., Sharma S., Kumar A. (2014). Making surrogate *β*-cells from mesenchymal stromal cells: perspectives and future endeavors. *International Journal of Biochemistry and Cell Biology*.

[176] Paek H. J. (2014). Adipose stem cell-based regenerative medicine for reversal of diabetic hyperglycemia. *World Journal of Diabetes*.

[177] Sensebé L., Bourin P., Tarte K. (2011). Good manufacturing practices production of mesenchymal stem/stromal cells. *Human Gene Therapy*.

[178] Tarte K., Gaillard J., Lataillade J.-J. (2010). Clinical-grade production of human mesenchymal stromal cells: occurrence of aneuploidy without transformation. *Blood*.

[179] Fiorina P., Jurewicz M., Augello A. (2009). Immunomodulatory function of bone marrow-derived mesenchymal stem cells in experimental autoimmune type 1 diabetes. *The Journal of Immunology*.

[180] Tolar J., Nauta A. J., Osborn M. J. (2007). Sarcoma derived from cultured mesenchymal stem cells. *Stem Cells*.

[181] Toma C., Pittenger M. F., Cahill K. S., Byrne B. J., Kessler P. D. (2002). Human mesenchymal stem cells differentiate to a cardiomyocyte phenotype in the adult murine heart. *Circulation*.

[182] Haque N., Rahman M. T., Abu Kasim N. H., Alabsi A. M. (2013). Hypoxic culture conditions as a solution for mesenchymal stem cell based regenerative therapy. *The Scientific World Journal*.

[183] Lennon D. P., Edmison J. M., Caplan A. I. (2001). Cultivation of rat marrow-derived mesenchymal stem cells in reduced oxygen tension: effects on in vitro and in vivo osteochondrogenesis. *Journal of Cellular Physiology*.

[184] Panchision D. M. (2009). The role of oxygen in regulating neural stem cells in development and disease. *Journal of Cellular Physiology*.

[185] Busuttil R. A., Rubio M., Dollé M. E. T., Campisi J., Vijg J. (2003). Oxygen accelerates the accumulation of mutations during the senescence and immortalization of murine cells in culture. *Aging cell*.

[186] Fehrer C., Brunauer R., Laschober G. (2007). Reduced oxygen tension attenuates differentiation capacity of human mesenchymal stem cells and prolongs their lifespan. *Aging Cell*.

[187] Stamati K., Mudera V., Cheema U. (2011). Evolution of oxygen utilization in multicellular organisms and implications for cell signalling in tissue engineering. *Journal of Tissue Engineering*.

[188] Liu J., Hao H., Xia L. (2015). Hypoxia pretreatment of bone marrow mesenchymal stem cells facilitates angiogenesis by improving the function of endothelial cells in diabetic rats with lower ischemia. *PLoS ONE*.

[189] Szokodi I., Tavi P., Földes G. (2002). Apelin, the novel endogenous ligand of the orphan receptor APJ, regulates cardiac contractility. *Circulation Research*.

[190] Kleinz M. J., Davenport A. P. (2005). Emerging roles of apelin in biology and medicine. *Pharmacology and Therapeutics*.

[191] Ishizuka T., Hinata T., Watanabe Y. (2011). Superoxide induced by a high-glucose concentration attenuates production of angiogenic growth factors in hypoxic mouse mesenchymal stem cells. *Journal of Endocrinology*.

[192] Mottaghi S., Larijani B., Sharifi A. M. (2012). Apelin 13: a novel approach to enhance efficacy of hypoxic preconditioned mesenchymal stem cells for cell therapy of diabetes. *Medical Hypotheses*.

[193] Zeng X., Yu S. P., Taylor T., Ogle M., Wei L. (2012). Protective effect of apelin on cultured rat bone marrow mesenchymal stem cells against apoptosis. *Stem Cell Research*.

[194] Eizirik D. L., Korbutt G. S., Hellerström C. (1992). Prolonged exposure of human pancreatic islets to high glucose concentrations in vitro impairs the *β*-cell function. *The Journal of Clinical Investigation*.

[195] Burger S. R. (2000). Design and operation of a current good manufacturing practices cell-engineering laboratory. *Cytotherapy*.

[196] http://ec.europa.eu/health/files/eudralex/vol1/dir_2001_83_consol_2012/dir_2001_83_cons_2012_en.pdf.

[197] http://ec.europa.eu/health/files/eudralex/vol-1/reg_2007_1394/reg_2007_1394_en.pdf.

[198] Halme D. G., Kessler D. A. (2006). FDA regulation of stem-cell-based therapies. *The New England Journal of Medicine*.

[199] Harvath L. (2000). Food and Drug Administration's proposed approach to regulation of hematopoietic stem/progenitor cell products for therapeutic use. *Transfusion Medicine Reviews*.

[200] Committee for Advanced Therapies Reflection paper on classification of advanced therapy medicinal products. http://www.ema.europa.eu/docs/en_GB/document_library/Scientific_guideline/2015/06/WC500187744.pdf.

[201] http://ec.europa.eu/health/files/eudralex/vol-1/reg_2007_1394/reg_2007_1394_en.pdf.

[202] Elseberg C. L., Leber J., Salzig D. (2012). Microcarrier-based expansion process for hMSCs with high vitality and undifferentiated characteristics. *International Journal of Artificial Organs*.

[203] Wuchter P., Bieback K., Schrezenmeier H. (2015). Standardization of Good Manufacturing Practice-compliant production of bone marrow-derived human mesenchymal stromal cells for immunotherapeutic applications. *Cytotherapy*.

[204] Salzig D., Leber J., Merkewitz K., Lange M. C., Köster N., Czermak P. Attachment, growth and detachment of human mesenchymal stem cells in a chemically defined medium. *Stem Cells International*.

